# Poor-Laying Hen Identification by Integrating HVI Color-Space Transformation and Differentiable Histogram

**DOI:** 10.3390/s26216730

**Published:** 2026-10-24

**Authors:** Jongwoong Seo, Seungwook Son, Sejun Lee, Munki Park, Jonggwan Kim, Jonghyeon Kim, Heechan Chae

**Affiliations:** Info Valley Korea Co., Ltd., Anyang 14067, Republic of Korea; seojongwoong@invako.kr (J.S.); sso7199@invako.kr (S.S.); lllss22@invako.kr (S.L.); mkpark@invako.kr (M.P.); jking@invako.kr (J.K.); jh.kim@invako.kr (J.K.)

**Keywords:** poor-laying hen identification, poultry monitoring, pigmentation analysis, color-space transformation, differentiable histogram layer

## Abstract

**Highlights:**

Head and leg images allow non-invasive observation of pigmentation changes associated with laying status. Motivated by this physiological relationship, this study combines HVI color-space transformation with differentiable histogram encoding of learned features for image-based screening of poor-laying hens.

**What are the main findings?**

- Motivated by pigmentation changes associated with laying status, CHIC combines HVI transformation and histogram features, improving classification over MobileNetV4-S baselines under frame-level evaluation.
- Histogram integration benefits both color and grayscale inputs, although its effects depend on the model configuration.

**What are the implications of the main findings?**

- The findings support physiologically motivated model design for image-based screening of poor-laying hens.
- Further validation of generalization to unseen cages and new housing conditions is needed for practical deployment.

**Abstract:**

Identifying poor-laying hens is important for reducing feed waste, but visual screening is labor-intensive. Motivated by the relationship between laying status and pigmentation, we propose CHIC (Color-Histogram Integrated Classifier), a lightweight model combining HVI (Horizontal, Vertical, Intensity) transformation with differentiable histogram encoding of learned feature activations and global-average-pooled features. Evaluation used a four-class head-and-leg patch dataset collected from 20 hens in six cages at a single facility, with cage-level laying-status labels. Across ten training runs with different development-data folds and a fixed frame-level test set, CHIC achieved mean binary F1-scores of 92.21% for head patches and 96.06% for leg patches, exceeding the MobileNetV4-S baselines. With 2.69 million parameters, the classifier had a mean forward-pass latency of 21.87 ms per patch on an NVIDIA Jetson Orin Nano Super. Component comparisons showed configuration-dependent effects; histogram integration also benefited grayscale inputs, while mean–standard-deviation pooling with HVI inputs achieved similar mean performance to CHIC. The classification advantage was maintained when predictions from detected head and leg regions were aggregated into hen-level decisions. However, performance declined substantially on unseen cages. These findings support CHIC as a candidate for automated poor-laying hen screening, with further validation required across independent hens and farms.

## 1. Introduction

The global egg industry constitutes one of the largest animal-protein sectors: world egg production reached 97 million tons in 2023 [1], and the global laying-hen population was estimated at 7.9 billion birds in 2020, of which 84.2% of commercial layers were housed in cage [2]. In this industry, laying rates are a key indicator that determines farm productivity and profitability. Laying hens continuously consume feed throughout the production period; therefore, when the laying performance of individual hens decreases, egg output declines relative to feed cost, directly leading to economic losses for farms. In particular, feed cost accounts for the largest proportion of production costs in laying hen systems, and previous studies have reported that approximately 60–70% of the total production cost in laying hen or egg production systems is associated with feed expenditure [3,4,5]. Accordingly, early identification and management of poor-laying hens, which consume feed at levels comparable to those of laying hens but contribute little or very little to egg production, is an important management factor for reducing feed resource waste and improving production efficiency in laying hen farms.

In conventional farms, poor-laying hens have mainly been identified through visual inspection or palpation by workers. Recent studies have also reported that identifying poor-laying hens using non-invasive and objective methods can contribute to improving the efficiency of laying hen production systems and optimizing resource utilization [6]. However, in large-scale laying hen farms, many individuals must be repeatedly inspected, making worker-dependent approaches limited in terms of time, labor, and consistency of judgment.

In precision livestock farming (PLF), computer vision has been widely investigated as a non-contact monitoring technology for automating labor-intensive observation tasks in livestock production environments. Previous studies have demonstrated its applicability to various livestock-monitoring tasks, including animal detection and counting, abnormal symptom detection, and robust monitoring under varying housing conditions [7,8,9,10,11]. However, poor-laying hen identification differs from general detection, counting, or behavior-recognition tasks. Its key visual evidence includes subtle external physiological cues associated with laying status, particularly differences in yellow pigmentation of the beak and shanks, as well as changes in comb color and morphology. These physiological cues motivate the use of both color and morphological information from head and leg images for poor-laying hen identification.

Among the external physiological cues associated with laying status, color change caused by pigmentation and depigmentation is particularly important. In actively laying hens, xanthophyll pigments in the body are used for yolk formation during sustained egg production, resulting in progressive depigmentation in the beak, face, and legs [12]. Previous studies have also reported a relationship between laying rate and the degree of pigmentation in the legs and beak, showing that yellow pigmentation on the body surface tends to decrease as laying continues [13]. In contrast, poor-laying hens may retain relatively strong yellow pigmentation in the beak, face, and legs because their reduced laying activity limits the transfer of pigments to the yolk [14]. Therefore, the head and leg regions are body parts where differences in pigment retention and depigmentation according to laying status can be observed relatively directly, and they can serve as major visual cues for poor-laying hen identification. Figure 1 compares representative external differences between laying hens and poor-laying hens in the head and leg regions. Xanthophyll-related bleaching of yellow pigmentation in the beak and shanks should be distinguished from changes in comb redness and size. Comb appearance is associated with vascular and hormonal factors, and actively laying hens generally have redder and fuller combs [6]. For the Hy-Line Brown hens used in this study, the expected visual cues therefore include differences in yellow pigmentation of the beak and shanks and in the redness and fullness of the comb.

Although the physiological relationship between laying status and pigmentation change is relatively well established, attempts to identify poor-laying hens based on color differences have not yet been sufficiently explored in laying hen image analysis. Existing poultry vision studies have mainly focused on macroscopic morphological or behavioral information, such as individual detection, dead bird detection, behavior tracking, and feather damage assessment. Recent deep learning-based studies have also been proposed for poor-laying hen identification; however, most of them rely on general RGB images and CNN-based classification structures, with limited structural consideration of how color information reflecting laying status should be separated from illumination and brightness variations [14]. In particular, the RGB color space contains chromatic and luminance information entangled within each channel, so actual pigmentation differences and simple brightness differences can be mixed under uneven illumination conditions inside poultry houses. This can hinder the model from consistently learning color differences associated with laying status.

In this study, motivated by the physiological relationship between laying status and pigmentation, we propose a lightweight classification model called CHIC (Color-Histogram Integrated Classifier) for identifying poor-laying hens from head and leg images. CHIC applies HVI (Horizontal, Vertical, Intensity) color-space transformation to represent chromatic components separately from intensity. A differentiable histogram layer encodes the distributions of learned feature activations extracted by the backbone. The resulting distributional descriptors are fused with global-average-pooled features to form a joint representation for classification.

The main contributions of this study are as follows.

- First, we propose CHIC, a lightweight architecture for poor-laying hen identification from head and leg images. CHIC combines an HVI input representation with differentiable histogram encoding learned feature activations and fuses the resulting distributional descriptors with global-average-pooled features.
- Second, we demonstrate higher mean F1-scores for both head and leg patches than the MobileNetV4-S baselines. Comparisons with component variants, alternative backbones, and mean–standard-deviation pooling characterize the performance gains of the proposed design and their dependence on the model configuration.
- Third, we extend the evaluation from individual image patches to hen-level decisions within a detection–classification pipeline by aggregating predictions from detected head and leg regions. We evaluate end-to-end performance at both region and hen levels and quantify the classifier’s computational requirements through parameter counts, FLOPs, and inference latency measured on an edge device.

## 2. Related Work

### 2.1. Computer Vision in the Poultry Industry

In the field of precision livestock farming, computer vision has been applied to a wide range of management tasks in poultry production, including dead-bird detection, behavior monitoring, welfare assessment, and individual tracking [15,16,17]. These tasks commonly target states that are visible as distinct objects, motions, or surface conditions in the image. Luo et al. [18] detected dead laying hens on a commercial farm using thermal infrared, near-infrared, and depth images. Subedi et al. [19] tracked feather-pecking behavior and the resulting plumage damage in cage-free housing using YOLOv5-based detectors. Lamping et al. [20] proposed ChickenNet, which extends Mask R-CNN to detect individual hens and simultaneously assess their plumage condition in commercial farms. Bist et al. [21] scored poultry footpad dermatitis using deep learning and thermal imaging. These studies demonstrate that camera-based monitoring of such visible states is feasible and accurate in poultry houses. However, the laying status targeted in the present study does not appear in the image as a distinct object, motion, or surface condition; it must be inferred from subtle cues in the external appearance of individual birds.

### 2.2. Color and Phenotypic Cues for Poor-Laying Hen Identification

A critical aspect of poor-laying hen identification is not merely the external appearance of body parts, but pigment-related color changes that occur according to laying status. Hens that continue laying normally show progressive depigmentation in the beak, face, and legs as body pigments are used for yolk formation [12,13]. In contrast, poor-laying hens may retain relatively strong yellow pigmentation because pigment transfer to the yolk is reduced. Therefore, color differences caused by pigment retention and depigmentation can serve as key physiological cues for identifying poor-laying hens.

Qin et al. [10] measured the comb spectra of hens with hyperspectral equipment under a halogen lamp, converted the spectral curves into two-dimensional feature maps using fast continuous wavelet transform, and classified them with a CNN, achieving 97.5% accuracy. These results indicate that laying status produces measurable spectral differences; however, this approach requires specialized sensing hardware and controlled illumination. Subsequent studies have addressed the task with standard RGB cameras. Qin et al. [22] compared CNN classifiers on 977 multi-view images of individual hens, in which ConvNeXt achieved 94.81% accuracy, and their heatmap-based attribution analysis indicated that the head region contributed most to the identification, followed by the feet, which supports the use of the head and leg regions in this study. Wang et al. [14] proposed a two-stage approach for stacked cage systems, in which an improved YOLOv8n segments the head regions of hens and a MobileNetV3 classifier identifies low-yield hens from the color and shape of the segmented heads, achieving 97.65% accuracy; however, only the head region is used, and the color features are extracted from the RGB representation as is. Chen et al. [23] combined adaptive brightness adjustment with a CNN-based detection structure for fine-grained recognition of caged-hen head states, showing that brightness correction can be important in caged-hen image analysis; however, this correction adjusts only the intensity level of the input.

In summary, these studies demonstrate that the discriminative information for laying status is concentrated in pigmented regions such as the head and legs, and that this information can be captured with standard cameras. However, in the existing RGB-based approaches, chromatic information is not separated from luminance, so pigmentation cues remain entangled with illumination effects [24], and the distributional characteristics of pigment retention and depigmentation are not explicitly modeled. In real farm images, stable extraction of pigmentation cues is further complicated by uneven illumination, cage structures, shadows, and changes in viewing angle. Therefore, more explicit separation of chromatic and luminance information is needed for stable learning of pigmentation-related cues in real farm images. Meeting this need through an explicit chromatic–luminance representation is one of the gaps that the present study aims to address.

### 2.3. Color-Space Representation and Histogram-Based Distribution Learning

Computer vision models generally use RGB images as input, but the RGB color space contains chromatic and luminance information entangled within each channel, making it sensitive to illumination variation [24]. As a result, actual pigmentation differences and simple brightness differences can be mixed within the model, which may degrade performance in poor-laying hen identification where color changes are important cues. In color-based object recognition and color constancy studies, the need for color representations robust to illumination, viewpoint, and geometric changes has long been discussed, and various color spaces and color representation methods have been applied to visual recognition problems. Cheng et al. [25] summarized the characteristics and limitations of various color-space representations in color image segmentation, while Gevers and Smeulders [26] and Geusebroek et al. [27] emphasized the importance of illumination-robust color-based recognition and color invariance.

Color spaces such as HSV have the advantage of separating hue, saturation, and value components compared with RGB. However, because hue has a circular structure, discontinuity can occur around 0° and 360°, where the same or similar colors are numerically represented as distant values. This characteristic may introduce unnecessary numerical distortion in neural networks that need to learn continuous color variation. Yan et al. [28] proposed the HVI color space for low-light image enhancement and introduced a polarized HS map and a learnable intensity function to alleviate the red discontinuity problem in HSV and the black noise problem in low-intensity regions. In this study, we use HVI not for low-light image enhancement, but as an input transformation method for separating chromatic and luminance information and representing pigmentation differences more stably in poor-laying hen identification.

While color-space transformation improves the stability of color representation, histogram-based representation summarizes the distribution of values within an image or feature map as a statistical representation. Traditional histograms have been widely used in image analysis because they intuitively represent the distributions of color, brightness, or intensity. However, hard-binning histograms are not differentiable and are therefore difficult to integrate into neural networks for end-to-end learning [29]. To address this issue, Wang et al. [30] proposed a learnable histogram layer that enables histogram features to be learned within deep neural networks and showed that bin centers and bin widths can be optimized through backpropagation. Peeples et al. [31] later introduced a localized histogram layer to compute local feature distributions within neural networks and applied it to texture and material recognition.

In this study, CHIC combines HVI color-space transformation with differentiable histogram encoding of learned backbone feature activations. The HVI transformation represents chromatic components separately from intensity, while the histogram layer summarizes the distributions of the extracted feature responses. These distributional descriptors are fused with global-average-pooled features to form a joint representation for poor-laying hen identification. This design is motivated by the physiological relationship between laying status and pigmentation in the head and leg regions.

## 3. Method

In this study, motivated by the physiological relationship between laying status and pigmentation, we propose CHIC for identifying poor-laying hens from head and leg images. CHIC applies an HVI color-space transformation to represent chromatic components separately from intensity and incorporates a differentiable histogram layer to encode distributions of learned backbone feature activations. Figure 2 shows the overall architecture of the proposed CHIC model. The input image is first transformed into the HVI color space and then passed through the backbone network. Global-average-pooled features and histogram-based distributional descriptors are then fused and fed into the final classifier.

### 3.1. Overview of the CHIC Architecture

CHIC is a lightweight image classification architecture that uses crop images of the head or leg regions of laying and poor-laying hens as input and classifies them into four classes: laying head, laying leg, poor-laying head, and poor-laying leg. In this study, the head and leg regions were selected because differences in pigment retention and depigmentation according to laying status are relatively apparent in these regions. CHIC is designed to stably learn these color-related cues.

The overall processing flow of CHIC can be summarized as follows. First, the RGB input image is transformed into the HVI color space and passed through the backbone network. Global average pooling is applied to the final backbone feature map, whereas the histogram branch processes the feature map from the selected backbone stage. The resulting histogram tensor is flattened and linearly projected, and the projected feature is concatenated with the global-average-pooled feature to form the final feature vector.

(1) $$X_{HVI}=T_{HVI}(X_{RGB}),F_{s}=f_{\theta }(X_{HVI}),z=[GAP(F_{s});P(Flatten\left(H\right))].$$

Here, $T_{HVI}(\cdot )$ denotes the HVI transformation function, $f_{\theta }(\cdot )$ denotes the backbone network, and $F_{s}$ denotes the final backbone feature map. H denotes the histogram tensor generated from the feature map at the selected backbone stage. GAP(⋅) represents global average pooling, Flatten(⋅) converts the histogram tensor into a vector, and P(⋅) denotes the linear projection.

The combined feature vector is then passed through a classifier to produce the final class probability:

(2) $$\hat{y}=softmax(g_{\phi }(z)).$$

where $g_{\phi }(\cdot )$ denotes the fully connected classifier and $\hat{y}$ represents the predicted class probability vector.

This architecture combines the global-average-pooled backbone representation with histogram-based descriptors of learned feature distributions. Global average pooling summarizes the mean response of each feature channel, while the histogram branch characterizes how feature activations are distributed within coarse spatial regions. These representations are fused for poor-laying hen identification.

### 3.2. HVI Color-Space Transformation

The key cues for poor-laying hen identification are differences in pigment retention and depigmentation associated with laying status. However, in RGB images, chromatic and luminance information are entangled within each channel. As a result, illumination changes inside poultry houses may be mixed with actual color differences. In this study, we apply the HVI color-space transformation proposed by Yan et al. [28] to the poor-laying hen identification task. The HVI transformation first converts an RGB image into the HSV color space and then re-represents the hue-saturation plane as horizontal and vertical chromatic components.

In HSV, the hue value has a circular structure, which can cause a red discontinuity problem around 0° and 360°, where similar colors are numerically represented as distant values. To alleviate this issue, the hue value is transformed into a polar-coordinate representation to compute the horizontal component h and the vertical component v:

(3) $$h=cos\left(2\pi H\right),v=sin\left(2\pi H\right).$$

where H denotes the hue channel in HSV. Equation (3) defines the hue-direction components, and the saturation component is incorporated later Equation (5).

In addition, HVI applies a learnable intensity collapse function to suppress unnecessary chromatic variation that can occur in low-intensity regions. The intensity collapse function is defined as

(4) $$C_{k}(x)={\left[sin\left(\frac{\pi I_{max}(x)}{2}\right)+\epsilon \right]}^{k}.$$

Here, k is a learnable parameter, ϵ is a small constant used to prevent gradient explosion, and $I_{max}$ denotes the intensity component of the input image. Equation (5) describes the process of constructing the final chromatic channels of HVI by combining the saturation S, the horizontal component h, the vertical component v, and $C_{k}$:

(5) $$\hat{H}=C_{k}⊙S⊙h,\hat{V}=C_{k}⊙S⊙v.$$

Finally, the input representation of CHIC is composed of the two chromatic channels and the intensity channel, as shown in Equation (6):

(6) $$X_{HVI}=[\hat{H},\hat{V},I_{max}].$$

Here, ⊙ denotes element-wise multiplication. Through this HVI transformation, chromatic and intensity components are more explicitly separated, thereby stabilizing RGB-based color representation that may be distorted by illumination changes. In this study, HVI is not used for low-light image enhancement, but as a color-space transformation to more stably represent differences in pigment retention and depigmentation between poor-laying and laying hens.

The overall transformation process is illustrated in Figure 3. In RGB space, chromatic and luminance information are jointly represented within the three color channels, making pigmentation cues sensitive to illumination variation. Although HSV separates hue, saturation, and value components, the circular nature of the hue channel can introduce discontinuity around the red region, where perceptually similar colors may be represented by numerically distant values. To alleviate this problem, the HVI transformation represents the hue–saturation plane as polarized horizontal and vertical chromatic components. In addition, the intensity collapse function reduces unstable chromatic responses in low-intensity regions, resulting in a more continuous and illumination-robust color representation. This property is useful for poor-laying hen identification because color differences in the head and leg regions should be considered separately from image brightness variation.

### 3.3. Differentiable Histogram Layer for Distribution Encoding

Although the HVI transformation stabilizes the color representation of the input image, the general spatial features extracted by the backbone may not sufficiently capture the distributional differences in pigment retention and depigmentation patterns that appear across the head or leg regions. The differences between poor-laying and laying hens often appear not as a single color value at a specific location but as distributional differences in color and intensity values across the region of interest. Therefore, CHIC incorporates a differentiable histogram layer into the backbone feature map to extract the distributional information of feature-map values in a learnable form.

A conventional histogram summarizes a distribution by assigning pixel or feature values to fixed bins. However, in a standard hard-binning scheme, bin membership is determined discretely, which results in zero gradients in most intervals or non-differentiable operations [32]. As illustrated in Figure 4, hard-binning assigns each value to a single discrete bin and produces stepwise responses that cannot propagate gradients, whereas soft-binning assigns continuous membership scores via a piecewise linear function, enabling differentiable histogram computation. Therefore, it is difficult to directly use hard-binning histograms inside neural networks for end-to-end learning. To address this limitation, we integrate a soft-binning-based differentiable histogram layer into CHIC based on the learnable histogram layer proposed by Wang et al. [30].

The histogram branch receives the feature map from the selected backbone stage. In the default configuration, the 480-channel Stage 4 feature map is reduced to 160 channels by a 1 × 1 convolution with a bias term, followed by batch normalization without learnable affine parameters. Soft binning is applied to the resulting reduced and normalized feature map, denoted by F in Equation (7). The bin centers are initialized from standard-normal quantiles, and the inverse-width parameters are learned in logarithmic form to ensure positivity. No normalization is applied across bins. Stage numbering follows the backbone feature-extraction indices shown in Figure 2.

Let the input feature map be $F\in R^{C\times H\times W}$, where $F_{c}(u,v)$ denotes the feature value at spatial location $\left(u,v\right)$ in the c-th channel. For each channel c and bin b, the differentiable histogram layer defines a learnable bin center $\mu_{c,b}$ and a scaling factor $\gamma_{c,b}$. The degree to which the feature value belongs to a bin is computed using the continuous membership score in Equation (7):

(7) $$E_{c,b}(u,v)=max\left(0,1-\gamma_{c,b}\;|F_{c}(u,v)-\mu_{c,b}|\right).$$

Here, $E_{c,b}(u,v)$ represents the degree to which $F_{c}(u,v)$ contributes to the b-th bin. $\mu_{c,b}$ denotes the center of the bin, and $\gamma_{c,b}$ is a scale parameter corresponding to the inverse of the bin width. This membership function is designed to assign a higher value when the feature value is closer to the bin center and to become zero when the distance exceeds a certain range. Therefore, unlike hard binning, it provides continuous gradients according to changes in feature values, and the bin parameters can also be learned through backpropagation.

The membership maps are first aggregated using local average pooling with a K×K window, followed by adaptive average pooling to a 2×2 output, as expressed in Equation (8)

(8) $$H_{c,b,r}=\frac{1}{\left|\Omega_{r}\right|}\sum_{p\in \Omega_{r}}\left[\frac{1}{\left|N_{K}\left(p\right)\right|}\sum_{q\in N_{K}\left(p\right)}E_{c,b}\left(q\right)\right],\;r=1,\ldots ,4.$$

Here, $N_{K}(p)$ denotes the valid positions within the K×K pooling window centered at p, and $\Omega_{r}$ denotes the adaptive pooling window corresponding to the r-th output cell. Local pooling uses a stride of 1 and padding of $\left\lfloor K/2\right\rfloor$, with padded positions excluded from the average. The kernel size K is set to 3 in the default configuration. The resulting histogram tensor H retains coarse spatial information through its 2×2 output and is flattened before linear projection. The computation pipeline of the differentiable histogram layer is illustrated in Figure 5.

### 3.4. Feature Fusion and Classification

In the final classification stage of CHIC, the spatial feature extracted from the backbone and the histogram feature extracted from the histogram layer are combined. The spatial feature obtained by applying global average pooling to the backbone feature map $F_{s}$ contains high-level semantic information summarized over the spatial dimensions. In contrast, the histogram feature H obtained from the differentiable histogram layer reflects the distributional characteristics of feature-map values.

In this study, the two features are concatenated and used as the input to the final classifier, as shown in Equation (9):

(9) $$z_{s}=GAP(F_{s}),z_{h}=P(Flatten(H)),z=[z_{s};z_{h}].$$

Here, $z_{s}$ denotes the global-average-pooled feature from the final backbone feature map $F_{s}$ and $z_{h}$ denotes the projected histogram feature. H is the histogram tensor obtained in Equation (8), and P(·) denotes a fully connected projection with 1280 input and output dimensions and a bias term. No activation or normalization is applied after this projection. During training, branch dropout was applied independently to each sample with a probability of 0.5. When applied, either the global-average-pooled feature vector or the projected histogram feature vector was selected with equal probability and set to zero before concatenation. Both feature vectors were used during standard inference.

(10) $$\hat{y}=softmax(Wz+b).$$

Here, W and b denote the weight matrix and bias of the fully connected classifier, respectively, and $\hat{y}$ represents the predicted class probability vector.

Through this structure, CHIC combines the global-average-pooled backbone representation with histogram-based descriptors of learned feature distributions. The fused representation is used to classify each input patch into one of four classes: laying head, laying leg, poor-laying head, and poor-laying leg. The backbone, histogram branch, linear projection, and classifier are jointly optimized during end-to-end training.

## 4. Experiments

### 4.1. Dataset

The dataset used in this study was constructed from 1794 original images collected in an experimental laying hen house at Chonnam National University. The hens were Hy-Line Brown layers aged 56 weeks at the time of image acquisition. Information on the xanthophyll content of the feed was not available. Six cages were used, three housing laying hens and three housing poor-laying hens; cages A01–A03 housed 2, 5, and 5 hens and cages B01–B03 housed 1, 4, and 3 hens (20 hens in total). Images were acquired by six fixed dome cameras, each dedicated to one cage and installed in front of it under the artificial lighting of the house: two Hanwha Wisenet QND-8023R cameras (cages A01 and B01, recorded at 2560 × 1440) and four XND-6020R cameras (cages A02, A03, B02, and B03, recorded at 1920 × 1080), so that images from both camera models were included in both laying-status groups. Both camera models were manufactured by Hanwha Vision Co., Ltd. (Seongnam, Republic of Korea). Camera settings were kept identical throughout the acquisition period without per-session adjustment, and no explicit colorimetric calibration, such as a color reference chart or cross-camera color matching, was performed. All images were acquired on the first day of the 28-day laying-performance observation period, and still images were extracted from the recordings at 60 s intervals (every 1800 frames at 30 fps), yielding the 1794 original images.

The original image set was divided into 1440 training, 177 validation, and 177 test images with the class ratio preserved, and images from both camera models were included in all subsets. The subsets were defined at the original-frame level before cropping, so all head and leg patches generated from a given frame were assigned to the same subset. However, this frame-level split did not ensure individual-hen or cage-level independence, because different frames could depict the same hens and cage environments. To evaluate performance on cages excluded from both training and validation, a separate three-fold cage-disjoint evaluation was conducted, as described in Section 4.4.6.

Since the objective of this study is not to localize hens in images but to classify poor-laying hens based on physiological pigmentation and depigmentation cues, we constructed an instance-level classification dataset by cropping the head and leg regions from the original images using bounding-box annotations. The head and leg regions were selected because pigmentation changes associated with laying status are visually apparent in these body regions. Each cropped patch was assigned a class label according to both laying status and body region. As a result, the final dataset consisted of four classes: laying head, laying leg, poor-laying head, and poor-laying leg. All cropped patch images were resized to 224 × 224 pixels to match the input resolution of the classification network. Representative patches of the four classes are shown in Figure 6.

The final classification dataset contained 10,523 patch instances, including 8443 training instances, 1022 validation instances, and 1058 test instances. The detailed dataset composition is summarized in Table 1.

The laying status of each hen was determined through a two-step verification procedure. First, candidate laying and poor-laying hens were initially screened by experienced poultry farm personnel in a laying hen house located in Sejong-si, Republic of Korea. This initial field screening considered practical assessment criteria for laying condition, including comb and wattle condition, body and abdominal condition, cloacal condition, plumage condition, and pigmentation-related traits. Although practical field assessment of laying condition involves multiple external phenotypic indicators, the present study focused on image-visible pigmentation-related cues in the head and leg regions. Accordingly, color and distribution information were treated as the primary visual cues for model development.

The selected hens were housed from the outset in separate cages according to their screened status, three cages of laying hens and three cages of poor-laying hens, in an experimental laying hen house, where laying performance was monitored through daily egg collection during a four-week observation period. Cage-level laying-performance records were used to verify the initial screening. A laying rate below 60% has been used to define low-production groups in previous research [33]. Xin et al. [34] also used this threshold to classify individual hens as low-laying based on daily egg-production records collected over four weeks. In our experiment, the four-week cage-level laying rate was calculated by dividing the total number of eggs collected over 28 days by the number of hens in the cage multiplied by 28. The laying rates were 67.86%, 75.00%, and 70.71% for cages A01, A02, and A03, respectively, and 0.00%, 38.39%, and 36.90% for cages B01, B02, and B03, respectively. Since egg production was recorded at the cage level, all hens within a cage share the cage-level label. Final laying-status labels were therefore assigned by combining the initial field screening by experienced poultry farm personnel with the subsequent cage-level laying-performance records. This procedure ensured that the dataset labels were not based solely on image-visible color cues but were supported by practical field screening and subsequent laying-performance records. Based on these labels, cropped head and leg patches were assigned to one of four classes: laying head, laying leg, poor-laying head, and poor-laying leg.

### 4.2. Experimental Setup and Implementation Details

Model training was performed using Python version 3.10.19 (Python Software Foundation, Beaverton, OR, USA), PyTorch version 2.10.0, CUDA 12.8, and cuDNN 9.10.2. The training hardware comprised an Intel Core i7-14700 CPU (Intel Corporation, Santa Clara, CA, USA) and a single NVIDIA RTX 4070 (12GB) GPU (NVIDIA Corporation, Santa Clara, CA, USA). MobileNetV4-S [35] with a width multiplier of 0.5, initialized with ImageNet-1K [36] pretrained weights, was adopted as the backbone for spatial feature extraction, and input images were resized and normalized to 224 × 224 resolution. Model training was performed for a total of 300 epochs using the AdamW optimizer (learning rate: 0.00025, weight decay: 0.005) with a cosine annealing scheduler. For training stability, 5 epochs of warmup and label smoothing of 0.1 were applied. The batch size was set to 16. Data processing, analysis, and visualization used NumPy version 2.2.6, Matplotlib version 3.10.8, OpenCV version 4.13.0 (OpenCV-python version 4.13.0.90), and Pillow version 12.1.0.

The same training hyperparameters were used across the color-space and backbone comparisons of CHIC, without separate tuning for individual color spaces or backbones. For each training run, the checkpoint achieving the highest validation top 1 accuracy was selected for test evaluation.

During training, random resized cropping was applied by sampling a crop covering 8–100% of the input patch area with an aspect ratio between 3/4 and 4/3. Each crop was resized to 224 × 224 using bilinear or bicubic interpolation selected at random. Random horizontal flipping was applied with a probability of 0.5. Color jitter was applied to brightness, contrast, and saturation, with an adjustment factor sampled from 0.6 to 1.4 for each attribute. No hue adjustment was applied.

FLOPs were calculated for a single 224 × 224 input, counting one multiply–accumulate operation as two FLOPs and including HVI and histogram operations. Model forward-pass latency was measured on an NVIDIA Jetson Orin Nano Super using FP16 AMP, batch size 1, and MAXN_SUPER mode without locking the clocks. The Jetson software environment comprised JetPack 6.2, PyTorch 2.5.0a0 (NVIDIA release 24.08), CUDA 12.6, and cuDNN 9.3.0. Latency is reported as mean ± standard deviation over ten measurements after warm-up.

### 4.3. Performance Evaluation

To evaluate the proposed CHIC architecture, Table 2 compares the MobileNetV4-S baselines with width multipliers of 0.5 and 1.0, the HVI-only model, and the full CHIC model. The region-specific binary F1-scores and ROC-AUCs for head and leg patches were used as the primary measures of laying-status discrimination. The overall four-class F1-score was also reported and was calculated as the unweighted mean of the four class-specific F1-scores (macro-F1). The training and validation frames were repartitioned into ten folds with the class ratios preserved. Each model was trained ten times using the settings described in Section 4.2, and each run was evaluated on the same fixed test set. Performance metrics are reported as the mean and standard deviation across these ten runs.

Table 2 shows that CHIC achieved the highest mean overall F1-score and the highest mean F1-scores for both head and leg patches among the evaluated configurations. HVI transformation alone increased the head F1-score but reduced the leg F1-score, yielding no improvement in mean overall F1-score. Adding the histogram branch improved F1-scores for both regions. CHIC also showed the lowest variability in overall F1-score across the development-data partitions.

The HVI transformation increased inference latency without changing the parameter count, while histogram integration increased both model size and computational cost. CHIC required fewer FLOPs than the width-1.0 baseline but exhibited longer inference latency on the evaluated device, indicating that a lower operation count did not necessarily translate into faster inference.

The additional parameters arise mainly from the histogram branch, whose distributional features are combined with the spatial features obtained through global average pooling. MobileNetV4-S was selected considering the computational constraints of poultry-house monitoring systems [37,38]. In the main CHIC configuration, the histogram layer was attached to Stage 4 with two bins and a kernel size of 3; the sensitivity to these settings is examined in Section 4.4.

Increasing the width of the MobileNetV4-S baseline substantially narrowed the performance gap with CHIC, indicating that greater backbone capacity also benefited classification. With a comparable parameter count, CHIC achieved higher mean overall and region-specific F1-scores, whereas the width-1.0 baseline achieved the highest head ROC-AUC.

Although the overall F1-score demonstrates the effectiveness of CHIC, it does not fully explain which types of classification errors were reduced by the proposed architecture. Therefore, we further analyzed the confusion matrices of MobileNetV4-S and CHIC on the test set to examine class-wise prediction patterns. In particular, this analysis focused on whether CHIC reduced confusion between laying and poor-laying classes and whether it improved discrimination between head and leg regions.

Figure 7 shows the confusion matrices of MobileNetV4-S and CHIC on the test set. MobileNetV4-S showed noticeable confusion between laying and poor-laying classes, particularly in the leg region. Specifically, 32 poor-laying leg samples were misclassified as laying leg samples, while 30 laying leg samples were misclassified as poor-laying leg samples. In addition, 28 poor-laying leg samples were misclassified as poor-laying head samples, indicating that the baseline had difficulty distinguishing both laying status and body region in some cases.

CHIC reduced these confusion patterns. The within-body-part leg confusions decreased from 62 to 22, and the number of correctly classified poor-laying leg samples increased from 268 to 315 out of 328. The cross-body-part confusion from poor-laying leg to poor-laying head was also eliminated. In the head region, CHIC showed smaller but consistent improvements, with correctly classified poor-laying head samples increasing from 137 to 144. These results indicate that CHIC improves class-wise discrimination by combining HVI-based spatial features with histogram-based distributional cues, especially for leg-region samples where pigmentation differences can be subtle.

To further examine the practical screening setting, the binary laying-status performance was evaluated separately for the head and leg regions, and the results are reported in Table 3. For each region type, the laying and poor-laying probabilities of the corresponding classes were compared, with poor-laying treated as the positive class, and a region was predicted as poor-laying when its conditional poor-laying probability was 0.5 or higher.

As shown in Table 3, CHIC improved laying-status classification in both head and leg patches, with larger gains in accuracy and recall for the leg region. The higher recall indicates a lower proportion of missed poor-laying patches at the selected threshold, while the increased ROC-AUC suggests better overall discrimination between laying and poor-laying classes.

To further examine the learned feature representations, we visualized the feature embeddings using t-SNE [39] implemented in scikit-learn version 1.7.2 and analyzed the class-discriminative regions using Grad-CAM [40].

Figure 8 shows the t-SNE embeddings of the feature representations extracted from MobileNetV4-S and CHIC. In MobileNetV4-S, the Laying head and Poor-laying head samples show considerable overlap, and the Laying leg and Poor-laying leg samples are also partially intermingled. This indicates that the baseline model has limited ability to separate laying status within the same body region. In contrast, CHIC forms more compact intra-class clusters and clearer inter-class boundaries across the four classes. These results suggest that the combination of HVI-based representation and histogram-based distribution encoding helps the model learn more discriminative feature representations for poor-laying hen identification.

Figure 9 shows the Grad-CAM visualizations for MobileNetV4-S and CHIC across the four classes. CHIC shows more concentrated activation within the head and leg regions, whereas MobileNetV4-S exhibits more diffuse responses extending into the surrounding areas. The spatial alignment between these activation maps and the segmented head or leg regions is quantified in Table 4.

Grad-CAM visualization and quantitative attention analysis were performed using the corresponding Stage 4 feature maps from the backbones of MobileNetV4-S and CHIC, each with dimensions of 480 × 7 × 7. For each test patch, gradients of the ground-truth class logit were computed with respect to these feature maps. In CHIC, the gradients included contributions from both the GAP-based classification path and the histogram–projection path. The resulting 7 × 7 Grad-CAM maps were bilinearly upsampled to 224 × 224. Quantitative evaluation was performed on all 1058 test patches, with the head or leg region of each patch segmented by prompting SAM3 [41] with the corresponding bounding box. SAM3 inference was performed using Transformers version 5.16.1 (Hugging Face, Inc., Brooklyn, NY, USA).

Four metrics were used to quantify attention alignment. Normalized RoI energy measures the proportion of attention energy within the segmented region relative to the area-based expectation for a spatially uniform map. Pointing game accuracy is the proportion of test patches in which the maximum of the attention map falls within the segmented region. Pixel ROC-AUC measures how well the attention scores distinguish pixels inside the region from those outside it. Normalized IoU@20 measures the overlap between the segmented region and the top 20% of pixels ranked by attention score, with correction for chance overlap.

As shown in Table 4, CHIC exhibited greater spatial alignment with the segmented head and leg regions than MobileNetV4-S. These attention-alignment metrics are reported as descriptive comparisons between models. Activation on the letterbox padding suggests that crop geometry may also contribute to the model responses. However, the native 7 × 7 resolution limits localization near anatomical and padding boundaries, and upsampling does not recover finer spatial detail. Moreover, anatomical masks may contain cage structures or residual background, so alignment with these masks does not establish which visual cues support the predictions. In particular, activation near the comb does not establish whether comb redness, morphology, or surrounding structures contributed to the prediction. These results therefore indicate spatial alignment with the anatomical regions without directly demonstrating the use of pigmentation information. Sensitivity to background and padding was further examined through background masking and padding removal experiments.

To further evaluate whether the effectiveness of the proposed CHIC architecture depends on a specific backbone, we applied the CHIC design to different lightweight and efficient backbone networks. In this experiment, MobileNetV4-S, EfficientNetV2-b0 [42], and GhostNetV3-100 [43] were used as backbone models. All backbone models were initialized with ImageNet-1K pretrained weights to ensure a fair comparison under the same pretraining setting. For each backbone, a CHIC variant was constructed by incorporating the HVI transformation, differentiable histogram layer, and feature-fusion design using the same histogram configuration as the main experiment (bin = 2, kernel = 3, stage = 4). This comparison was conducted to examine whether the proposed combination of HVI-based representation and histogram-based distribution encoding provides consistent performance gains across different feature extraction architectures.

As shown in Table 5, CHIC improved the mean F1-score of MobileNetV4-S, whereas the CHIC variants of EfficientNetV2-b0 and GhostNetV3-100 yielded lower mean F1-scores than their corresponding baselines. This pattern suggests that the benefit of combining HVI transformation with histogram encoding depends on the backbone. The higher baseline performance of the two alternative architectures may leave less room for incremental gains, although this alone does not explain the observed decreases. Another possible factor is the use of the same histogram bin count, kernel size, and attachment stage across all three backbones. The configuration used in the main MobileNetV4-S experiment may not be equally suited to the feature representations of the other architectures, potentially limiting the effectiveness of histogram integration and feature fusion. These results therefore support CHIC’s effectiveness with the tested MobileNetV4-S configuration, while suggesting that its extension to other backbones requires consideration of the compatibility between the backbone features and the histogram configuration. The EfficientNetV2-b0 and GhostNetV3-100 baselines achieved higher mean F1-scores than CHIC with MobileNetV4-S but required more parameters, FLOPs, and inference time on the evaluated device. Incorporating CHIC increased computational costs for all three backbones, while an improvement in mean F1-score was observed only for MobileNetV4-S. The GhostNetV3-100 timings were obtained without deployment reparameterization.

### 4.4. Ablation Study

To analyze the contribution and stability of the proposed CHIC architecture, we conducted a series of ablation and sensitivity experiments. First, component-level ablation was performed to separately examine the effects of the HVI transformation and the differentiable histogram layer. Second, illumination-related robustness was evaluated by applying brightness, contrast, and gamma transformations to the test images after training [44,45]. Third, saturation sensitivity analysis was conducted to investigate how the models respond when chromatic information is progressively weakened. Finally, the configuration of the differentiable histogram layer was analyzed by varying the connected backbone stage, kernel size, and number of histogram bins. These experiments were designed to verify whether the proposed HVI transformation and histogram-based distribution encoding contribute to stable poor-laying hen identification under color and illumination variations.

#### 4.4.1. Component-Level Ablation and Comparison of Color Spaces

To isolate the contribution of each component in the proposed CHIC architecture, we conducted a component-level ablation study using MobileNetV4-S as the baseline backbone. Four configurations were compared: the RGB-based MobileNetV4-S baseline, the baseline with only the HVI transformation, the baseline with only the differentiable histogram layer, and the full CHIC model combining both the HVI transformation and histogram-based distribution encoding. In addition, HSV, YCbCr, and CIELAB were included as alternative chromatic–luminance representations, each evaluated with and without the histogram layer under the same configuration. Models with and without the histogram layer were also trained and evaluated using grayscale inputs to assess performance in the absence of chromatic information.

The RGB input with the histogram layer was included to examine whether histogram-based distribution feature encoding alone can improve classification performance without the HVI color-space transformation, whereas the full CHIC model was used to evaluate the combined effect of color-space separation and histogram-based distribution learning. In Table 6, Color Space denotes the input representation, Histogram indicates whether the differentiable histogram layer is applied, and Bin, Kernel, and Stage denote the histogram-layer configuration; “–” indicates that the corresponding component is not applied.

As shown in Table 6, adding the histogram branch improved the mean F1-score for every input representation, whereas differences among the color representations without the branch were relatively small. This pattern is consistent with histogram features providing distributional information that complements the spatial features learned by the backbone. HVI achieved the highest mean F1-score without the histogram branch, but CIELAB achieved the highest mean after its addition, followed by YCbCr and HVI. The change in ranking and the small differences among the leading configurations suggest that the effectiveness of a color representation should be considered together with the feature-encoding architecture, rather than attributed to an inherent advantage of HVI.

The grayscale configuration also benefited from histogram integration, suggesting that the observed improvement was not limited to chromatic information. One plausible explanation is that the branch can encode distributions of features associated with intensity or texture, in addition to color-related features. Nevertheless, grayscale performance remained below that of the corresponding color-input configurations, supporting the usefulness of color information in the tested setting. Because histogram integration also increased the parameter count, this comparison alone does not separate the contribution of distribution from that of increased model capacity.

#### 4.4.2. Robustness to Illumination-Related Perturbations

To evaluate robustness to illumination-related variations, we conducted additional tests by applying brightness, contrast, and gamma transformations to the test images after training. Brightness variation changes the overall image intensity, contrast variation adjusts the difference between bright and dark regions, and gamma correction modifies the nonlinear luminance distribution. For each transformation, factor values from 0.2 to 2.0 were applied in steps of 0.2, while the training procedure and model weights remained unchanged. The same perturbation settings were applied to the RGB baseline, HVI-only, RGB-histogram, and CHIC models. Table 7 reports the mean F1-score across the perturbation factors for each transformation, excluding the original condition. Average drop denotes the mean decrease in F1-score from the original condition across the brightness, contrast, and gamma transformations.

As shown in Table 7, HVI-only exhibited a smaller average performance drop than the RGB baseline, while both histogram-based configurations achieved higher mean F1-scores across all three perturbation types. CHIC and RGB-histogram showed similar average drops, with RGB-histogram achieving higher mean F1-scores under brightness and contrast perturbations and CHIC performing better under gamma perturbations. These results suggest that the contribution of HVI to robustness depends on the type of perturbation and its combination with the histogram branch. The improvement over the RGB baseline therefore cannot be attributed solely to the HVI transformation.

#### 4.4.3. Chromatic Sensitivity Analysis

Two experiments were conducted to examine sensitivity to chromatic perturbations: saturation reduction and simulated correlated color temperature (CCT) shifts. Four MobileNetV4-S configurations were evaluated: the RGB baseline, HVI-only, RGB + Histogram, and CHIC. In the saturation experiment, the saturation factor of the test images was varied from 0.0 to 1.0 in increments of 0.1, where 0.0 represents grayscale and 1.0 represents the original input. The models were evaluated under these perturbations without retraining.

As shown in Figure 10a, classification performance generally declined as saturation decreased, with more pronounced losses at low saturation levels. RGB + Histogram retained the highest mean F1-score under strong desaturation, whereas CHIC and RGB + Histogram showed similar performance at moderate and high saturation levels. HVI-only did not consistently outperform the RGB baseline. These results suggest that sensitivity to saturation reduction depends on the combination of the input representation and histogram-based feature encoding.

Because these models were trained on color inputs, test-time desaturation changes both the available chromatic information and the input distribution. The observed degradation therefore characterizes the sensitivity of the trained models to this change. The grayscale-trained models in Table 6 provide complementary evidence: histogram integration improved classification performance even without chromatic information, while the corresponding color-input configurations achieved higher mean F1-scores. These findings support the usefulness of color information in the tested setting while indicating that the benefit of histogram integration is not limited to chromatic features.

To further examine sensitivity to chromaticity changes that are not captured by the intensity-level perturbations in Section 4.4.2, we additionally simulated CCT shifts on the test images. Each image was linearized from sRGB to CIE XYZ, chromatically adapted to a target white point on the Planckian locus using the Bradford transform, and re-encoded to sRGB while preserving the mean CIE Y so that only chromaticity, not intensity, was altered. Because the images were captured with the camera’s white balance and stored in sRGB, whose reference white is D65, the untransformed test images were treated as the 6500 K reference; the simulated CCT values therefore denote the target white point relative to this reference rather than the physical CCT of the house lighting. Target CCTs of 2700, 3500, 4500, 5500, 7500, and 8500 K were evaluated without retraining the models, and no white-balance correction was applied after the transformation, so the 2700 K condition represents an extreme warm-cast stress test.

As shown in Figure 10b, all models experienced substantial performance degradation under strong shifts from the reference white point, particularly at 2700 K. CHIC achieved the highest mean F1-score at the warmest target, whereas RGB + Histogram performed better at several other target white points. CHIC also performed below the RGB baseline at 3500 and 4500 K, indicating that its relative performance depended on the simulated CCT. None of the evaluated configurations was robust to strong uncorrected chromaticity shifts. Practical deployment under substantially different lighting conditions therefore requires appropriate color calibration and white-balance control. The simulation approximates chromaticity changes in the sRGB domain and does not reproduce the full spectral characteristics of physical light sources.

#### 4.4.4. Sensitivity Analysis of Histogram-Layer Configuration

To further examine whether the performance gain of CHIC depends on a specific histogram-layer configuration, we conducted a sensitivity analysis by varying the backbone stage to which the histogram layer was attached and the corresponding kernel size. The number of bins was fixed to 2, following the main CHIC configuration. This experiment evaluates whether the proposed histogram branch maintains stable performance across different feature extraction levels and kernel-size settings. The trained CHIC models were additionally evaluated using only the average-pooled features or only the histogram features, without retraining. In Table 8, F1-score denotes the result obtained by fusing both features, whereas Average Pooling and Histogram Pooling report the F1-scores obtained using each feature branch alone.

As shown in Table 8, the mean F1-score of the full CHIC model remained within a relatively narrow range across the tested configurations, with the highest mean observed at Stage 4. This indicates limited variation across the examined combinations of attachment stage and kernel size. However, because these two settings were varied together, their individual effects cannot be isolated from this comparison.

Evaluating the trained models with one feature branch at a time revealed differences in their reliance on the two representations. For the earlier attachment configurations, retaining only the histogram features preserved performance better than retaining only the average-pooled features, suggesting greater reliance on the histogram branch within the jointly trained model. The gap between the two evaluations narrowed at later stages. One possible explanation is that the two branches provide more overlapping task-relevant information in these configurations, reducing their complementarity. At Stage 4, either feature branch alone yielded a slightly higher mean F1-score than the full model, indicating that fusion did not provide an additional gain in this setting. These observations characterize the use of jointly learned features and suggest that their complementary contribution depends on the configuration.

After analyzing the stage and kernel-size configurations, we further investigated the effect of the number of histogram bins, with the histogram layer attached to Stage 4 and the kernel size fixed at 3. The histogram descriptor was maintained at 1280 dimensions to match the global-average-pooled feature. With a fixed 2 × 2 spatial grid, increasing the number of bins was accompanied by a corresponding reduction in the number of channels, keeping the total histogram descriptor size unchanged.

As shown in Table 9, both the mean validation and test F1-scores varied within relatively narrow ranges across the evaluated bin counts. Sixteen bins yielded the highest mean validation F1-score, whereas four bins yielded the highest mean test F1-score. Increasing the number of bins did not produce a consistent improvement across the two sets.

Under the fixed descriptor dimensionality, increasing the number of bins provides more distributional responses per channel but requires a corresponding reduction in the number of channels. The potential benefit of additional bins may therefore be offset by the narrower channel representation, which could explain the limited variation in performance. The accompanying decrease in parameter count results from the narrower channel-reduction layer. These comparisons reflect the combined effects of bin count and channel dimensionality.

#### 4.4.5. Comparison with Statistical Pooling

To examine whether the differentiable histogram layer can be replaced by a simpler statistical summary of the feature distribution, the histogram branch of CHIC was replaced by mean–standard-deviation pooling, in which the channel-wise mean and standard deviation of the Stage 4 feature map are concatenated with the global-average-pooled feature. This variant was trained for each of the five input color spaces with the configuration of Section 4.2. Table 10 reports the overall F1-score and the region-specific F1-scores and ROC-AUCs for head and leg patches.

As shown in Table 10, mean–standard-deviation pooling achieved classification performance close to that of CHIC. The most direct comparison is between HVI-Mean-Std and CHIC, which use the same input representation but different summaries of the feature distribution. Their mean overall F1-scores were close, and the region-specific metrics showed no consistent advantage for either variant. RGB-Mean-Std achieved the highest mean overall F1-score among the evaluated configurations, although comparisons across different color spaces also reflect differences in the input representation.

A plausible explanation is that the channel-wise mean and standard deviation already retain much of the information needed for classification by summarizing the average activation level and its dispersion within each feature channel. Under the tested conditions, representing these distributions with learnable histogram bins may therefore provide limited additional discriminative information. These results support mean–standard-deviation pooling as a viable alternative for the classification task evaluated here, while leaving the additional benefit of histogram encoding unestablished by these classification metrics alone.

#### 4.4.6. Generalization to Unseen Cages

To evaluate transfer beyond the cages represented during training, we conducted a three-fold cage-disjoint evaluation using the 10,523 padded head and leg patches. Each fold held out a pair of cages for testing: A01 and B01 in Fold 1, A02 and B02 in Fold 2, and A03 and B03 in Fold 3. All patches from the held-out cages were excluded from both training and validation.

Within the remaining four cages, training and validation data were separated at the recording session level. A session was defined as a group of frames associated with the same camera, cage, and recording start time. Session assignments were fixed using seed 42 and stratified by cage and recording date, yielding approximately an 80:20 training-to-validation split by patch count. No recording session or original frame was shared between training and validation. Validation therefore assessed performance within the cages represented during training, whereas testing assessed transfer to the held-out cages.

As shown in Table 11, all models exhibited substantially lower test F1-scores than validation F1-scores. CHIC achieved the highest mean test F1-score among the compared models, but its performance decreased from 94.82% on in-cage validation data to 60.51% on unseen cages. The region-specific results also varied considerably across the held-out cage pairs, particularly for leg patches. These findings indicate that the strong performance observed when training and evaluation data shared the same cages was not maintained under cage-disjoint testing.

A possible explanation is that the models learned a combination of laying-status cues and cage-associated visual information that did not transfer consistently to new cages. Differences in hen appearance, backgrounds, camera viewpoints, and camera characteristics may contribute to this gap. In particular, the A01–B01 fold also held out the only cages recorded with the QND-8023R cameras, introducing a camera-model change alongside the cage change. These factors were not isolated in the present experiment, so the performance decrease cannot be attributed to a single cause. Although CHIC maintained the highest mean test performance, the results reveal a substantial limitation in generalization to unseen cages.

#### 4.4.7. Analysis of Cage Identity and Input Perturbations

To examine cage-associated information and sensitivity to visual context, we conducted three diagnostic analyses using the five-seed classification checkpoints from the original padded 8:1:1 frame-level split. The classification networks remained fixed throughout these analyses. First, a separate linear probe was trained to predict the six cage labels from the features supplied to each model’s final classifier. All 10,523 patches were included, with recording sessions divided into training, validation, and test sets using a 60:20:20 split for the probe. Cage prediction accuracy was measured on sessions held out from probe training.

Second, background removal was performed by setting pixels outside the SAM-derived hen mask to black while leaving the letterbox padding unchanged. Third, padding removal was performed by cropping the letterbox bands from the normalized input tensors using content-box coordinates mapped through the standard resizing and center-cropping operations. The remaining content was supplied to the network at a batch size of 1 without resizing or additional padding. These inputs retained approximately 56% of the original input-window area on average, with a minimum crop dimension of 8 pixels enforced. The background and padding removal tests were performed separately on the same 1058 test patches.

As shown in Table 12, cage identity was predictable from the learned features of all four models, with the histogram-augmented models yielding higher probe accuracies than their corresponding counterparts without the histogram branch. This indicates that cage-associated information remained accessible in the learned representations. Such information may originate from cage backgrounds, camera characteristics, or the appearances of hens associated with each cage. However, successful cage prediction by a separate probe does not establish that the laying-status classifier used cage identity as a basis for its decisions.

Background removal reduced classification performance more than padding removal for all models, while CHIC retained the highest mean F1-score under both conditions. A possible explanation is that background regions contribute to the feature statistics learned from the original images, making the classifiers sensitive to their removal. Nevertheless, background masking also introduces altered image statistics and mask boundaries. Similarly, although padding removal preserved the scale of the retained image content, it changed the spatial extent of the feature maps and the regions aggregated by pooling. The observed performance losses therefore reflect sensitivity to both the removed context and the resulting changes in input conditions. These findings are consistent with cage-associated visual information contributing to the generalization gap, but they do not isolate its causal contribution.

#### 4.4.8. Classification Using Handcrafted Color Features

To examine the classification performance of simple color statistics, we extracted color moments and color histograms from RGB, HSV, CIELAB, YCbCr, and HVI representations, with the HVI parameter k fixed at 0.4. Color moments comprised the mean, standard deviation, and skewness of each channel, yielding a nine-dimensional descriptor. Color histograms used 16 bins per channel, normalized by the number of selected pixels, yielding a 48-dimensional descriptor.

Features were computed using three pixel subsets: the entire padded crop, the pixels within the content box, and the pixels within the intersection of the SAM-derived head or leg mask and the content box. Thus, the background-excluded condition omitted both background and padding pixels from feature computation. For each combination of descriptor, color space, and pixel subset, a random forest classifier with 300 trees (scikit-learn version 1.7.2) was trained separately using the 8443 training patches from the original 8:1:1 split. Each classifier was evaluated on the same 1058 test patches using the corresponding feature-extraction condition. The validation subset was not used for classifier training or hyperparameter selection.

As shown in Table 13, color histograms achieved higher mean F1-scores than color moments across all tested color spaces and pixel-selection conditions. This suggests that a more detailed representation of the color distribution may provide useful information beyond a small set of summary statistics, although the difference in descriptor dimensionality should also be considered. The relative performance of the color spaces depended on the descriptor and the selected pixels. HVI performed best for color moments with and without padding, whereas HSV performed best for color histograms under these two conditions. Thus, the results do not indicate a consistent advantage for a single color space across all configurations.

Excluding padding generally improved classification performance, particularly for RGB and HSV color moments. One possible explanation is that repeated padding pixels influence the global color statistics, while restricting feature computation to the content region reduces this influence. Further excluding the background did not consistently improve performance, suggesting that restricting the descriptors to segmented head and leg pixels does not necessarily produce more discriminative color statistics. This may reflect both the loss of class-associated information outside the segmented regions and changes in the sampled color distribution. These comparisons concern classifiers trained separately for each pixel subset, whereas Table 12 evaluates fixed CNNs under modified test inputs. Although the handcrafted descriptors provided useful classification information, their highest mean F1-score remained below that of CHIC on the same frame-level split.

#### 4.4.9. Comparison with Previous Studies

To compare CHIC with classification models adopted in previous studies, MobileNetV3-S [14], ConvNeXt-Tiny [22], and ConvNeXt-Tiny with squeeze-and-excitation (SE) [22] were evaluated using the same training, validation, and test split as CHIC. The training settings for the comparison models followed their respective studies, while CHIC was trained using the configuration described in Section 4.2. Classification performance was assessed using the overall F1-score and region-specific F1-scores for head and leg patches. Parameter counts, FLOPs, and inference latency were also compared alongside classification performance.

As shown in Table 14, CHIC achieved the highest mean overall F1-score among the methods evaluated on the present dataset, including those adopted from previous studies. The improvement was observed for both head and leg patches, indicating that the performance advantage was not limited to a single anatomical region. Among the methods from previous studies, ConvNeXt-Tiny achieved the highest mean overall F1-score, while its SE-equipped variant showed lower performance, particularly for leg patches.

MobileNetV3-S had the smallest parameter count and the shortest inference latency among the compared models, but its mean F1-scores were lower than those of CHIC. CHIC used substantially fewer parameters than the ConvNeXt-based models and achieved higher mean F1-scores for both head and leg patches. Its inference latency was higher than that of ConvNeXt-Tiny but lower than that of the SE-equipped variant.

### 4.5. End-to-End Pipeline Evaluation

To evaluate classification performance under automatic localization, the full detection and classification pipeline was assessed on 177 test frames containing 1058 annotated head and leg regions and 462 annotated whole-hen boxes. Both pipelines used the same YOLO26n detector [46], combined with either MobileNetV4-S with a width multiplier of 0.5 or CHIC. The YOLO26n detector was trained using Ultralytics version 8.4.33 (Ultralytics Inc., Frederick, MD, USA). The classifiers trained with random seed 42 were used for this evaluation. Detected head and leg regions were cropped and passed to the classifier. For each crop, the laying and poor-laying probabilities corresponding to the detected region type were renormalized to obtain conditional laying-status probabilities.

At the region level, an annotated region was counted as an end-to-end success when it was localized at an IoU of at least 0.5 and its four-class label was correctly predicted. Regions missed by the detector were counted as failures. The region-level success rate was evaluated at a detector confidence threshold of 0.25 and an NMS IoU threshold of 0.45. Joint mAP@0.5 and mAP@0.5:0.95 evaluated the pipeline as a four-class detector, using a confidence sweep starting at 0.001 and an NMS IoU threshold of 0.7. The confidence of each four-class prediction was calculated as the product of the detector confidence and the conditional classifier probability.

At the hen level, each detected head or leg region was assigned to the smallest detected whole-hen box containing its center. The conditional poor-laying probabilities of the assigned regions were averaged to obtain a hen-level score, with a threshold of 0.5 used to classify the hen box as poor-laying. Hen-level E2E success required both localization at an IoU of at least 0.5 and correct laying-status classification, with all 462 annotated hen boxes included in the denominator. Hen-level classification accuracy was calculated over the 371 localized hen boxes with at least one assigned head or leg region.

As shown in Table 15, the pipeline incorporating CHIC achieved a higher region-level E2E success rate and higher joint mAP scores than the pipeline using MobileNetV4-S. CHIC also achieved higher hen-level E2E success and classification accuracy, showing that its performance advantage was maintained when head and leg predictions were aggregated into a hen-level decision. Since both pipelines used the same detector, these differences reflect the effect of the classifier and its resulting laying-status predictions.

For CHIC, hen-level classification accuracy reached 98.38% among localized hen boxes with assigned regions, whereas the hen-level E2E success rate was 79.00% over all annotated hen boxes. This difference reflects the inclusion of missed hen detections and hen boxes without assigned regions in the E2E evaluation. Improving hen detection and part assignment is therefore important for translating the high classification accuracy into a higher end-to-end success rate.

## 5. Discussion

The experimental results can be interpreted in the context of previously reported approaches to poor-laying hen identification. Existing vision-based studies have addressed this task either as RGB-based appearance classification with generic CNN architectures [14,22] or through specialized sensing hardware such as hyperspectral imaging [10]. Wang et al. [14] combined YOLOv8n-based head detection with a MobileNetV3 classifier in stacked cage systems, and Qin et al. [22] showed through attribution analysis that the head region contributes most to identification; however, in both cases, pigmentation-related color information was processed implicitly within standard RGB pipelines. In the present study, CHIC combined HVI transformation with differentiable histogram encoding and improved laying-status discrimination for both head and leg patches relative to the width-0.5 MobileNetV4-S baseline. The performance gap narrowed when the baseline width was increased, indicating that model capacity should also be considered when interpreting this improvement. The chromatic sensitivity analysis and grayscale-trained comparisons support the usefulness of color information, while the improvement from histogram integration with grayscale inputs indicates that its benefit is not limited to chromatic features. The quantitative attention analysis (Table 4) showed greater spatial alignment with the segmented head and leg regions but did not identify the specific visual cues responsible for classification. The architecture was motivated by the established poultry-science finding that xanthophyll transfer to the yolk produces progressive depigmentation of the beak, face, and legs during sustained laying [12,13], although the present analyses do not establish that the classifier specifically learned this physiological mechanism.

The contribution of this study can be considered in three aspects. First, whereas the physiological relationship between laying status and pigmentation has long been documented [12,13] and vision-based identification has recently been demonstrated [10,14,22], this study incorporates that physiological motivation into a classification architecture combining color transformation and feature-distribution encoding. Second, the HVI color space, originally proposed for low-light image enhancement [28], is repurposed here as a physiologically motivated input representation, extending its applicability beyond image restoration. Third, the differentiable histogram layer, previously applied mainly to texture and material recognition [30,31], is used here to encode distributions of learned feature values in livestock imagery. The component comparisons nevertheless showed configuration-dependent benefits: HVI alone was not consistently superior, mean–standard-deviation pooling with HVI inputs achieved performance close to CHIC, and CHIC integration did not improve every backbone. The sensitivity analyses (Table 8 and Table 9) also involved coupled changes in attachment stage and kernel size or in bin count and channel dimension. These analyses characterize the reference design without establishing two bins as the optimal setting.

From a practical perspective, the proposed approach uses commodity RGB cameras and a lightweight model with 2.69M parameters for poultry-house monitoring [37,38]. CHIC achieved a mean forward-pass latency of 21.87 ms per patch on an NVIDIA Jetson Orin Nano Super. Figure 11 illustrates how the proposed classifier would be deployed in a commercial caged house. Images captured by fixed cameras in front of the cage rows are processed by a lightweight detector that localizes hens and their head and leg regions; the localized regions are cropped and resized into patches, which CHIC classifies into per-patch laying and poor-laying probabilities. Patch-level conditional poor-laying probabilities assigned to the same detected hen box are then aggregated into a hen-level screening score, and detected hens exceeding a threshold are flagged for manual verification, so that inspection labor is concentrated on a small number of candidate hens rather than the entire flock. The feasibility of this pipeline was examined in Section 4.5: CHIC retained its advantage over the baseline at both the region and hen-box levels when the same detector was used. Classification accuracy among localized hens with assigned regions was higher than the end-to-end success rate over all annotated hens, reflecting the additional failures caused by missed detections and unsuccessful part assignments. The hen-level aggregation is a simple averaging of conditional poor-laying probabilities, and its screening threshold should be tuned using validation data to the false-alarm tolerance of each farm.

Several limitations of the present findings should be acknowledged. First, laying-status labels were verified at the cage level using group production-rate criteria; although the labeling procedure combined field screening by experienced personnel with subsequent laying-performance records, within-cage individual variation cannot be captured under cage-level recording. The initial screening included pigmentation-related traits, so the study population was partly selected using the visual cues under investigation. Consequently, the classification results cannot be regarded as independent validation of pigmentation-related physiological mechanisms. Second, all images were collected in a single experimental laying hen house on a single day using six fixed cameras, and because the same hens appear in multiple frames, the image-level split does not guarantee subject-level independence. Although the split was performed at the frame level with both camera models included in all subsets, and the cross-validation analysis in Section 4.3 confirmed that the performance is stable across different partitions of the development data, the cage-disjoint evaluation revealed a substantial decline in performance on previously unseen cages. The cage-identity probe and input perturbation experiments further highlighted possible sensitivity to cage-associated information and visual context, although they do not isolate the causes of this transfer gap. Generalization to unseen individuals, farms, and acquisition conditions remains to be validated; multi-session acquisition with individual-level partitioning and independently verified individual laying records are required for this purpose. Third, the illumination-related analyses in Section 4.4.2 and Section 4.4.3 rely on synthetic perturbations; in particular, the CCT simulation approximates chromaticity shifts in the sRGB domain rather than reproducing the spectral power distribution of real light sources, and no white-balance correction was applied, so acquisition under different physical illuminants with colorimetric calibration is required for a rigorous evaluation. All evaluated models remained vulnerable to strong uncorrected chromaticity shifts, reinforcing the need for white-balance control. Fourth, the evaluation was limited to a single breed, and pigmentation dynamics may differ across breeds with different skin and plumage characteristics. Finally, the end-to-end evaluation showed that detection coverage and part assignment are important factors limiting the full-pipeline performance, and detection quality under occlusion and pose variation in commercial houses will directly affect the achievable screening performance; improving the detector is outside the scope of this study. These limitations define the boundary conditions of the current findings.

## 6. Conclusions

This study proposed CHIC, a lightweight image classification architecture for poor-laying hen identification. The proposed approach was motivated by the physiological relationship between laying status and pigmentation patterns observed in the head and leg regions of laying hens. CHIC was designed to combine color-space transformation with explicit encoding of learned feature distributions for distinguishing poor-laying hens from laying hens.

CHIC integrates two components. First, the HVI color-space transformation separates chromatic components from the intensity component. Second, the differentiable histogram layer encodes distributional characteristics of features extracted from HVI-transformed inputs for fusion with the global-average-pooled representation. By combining spatial features from a lightweight backbone with histogram-based distribution features, CHIC forms a joint representation for poor-laying hen identification.

Experimental results demonstrated the effectiveness of the proposed architecture. The MobileNetV4-S-based CHIC model improved laying-status discrimination for both head and leg patches relative to the width-0.5 MobileNetV4-S baseline, with 2.69M parameters and a mean forward-pass latency of 21.87 ms per patch on an NVIDIA Jetson Orin Nano Super. The component-level ablation study showed that the benefits of HVI transformation and histogram integration depended on the configuration and mean–standard-deviation pooling, with HVI inputs achieving performance close to CHIC. In addition, the cross-backbone comparison showed that the improvements observed with MobileNetV4-S did not extend to all tested backbones.

Further analyses examined the prediction patterns and robustness of CHIC. Confusion matrix and t-SNE analyses supported improved class discrimination, while Grad-CAM and quantitative attention analysis showed greater spatial alignment with the head and leg regions without identifying the specific visual cues responsible for classification. The illumination robustness analysis indicated that CHIC maintained more stable performance under brightness-, contrast-, and gamma-related variations than the RGB baseline. The saturation sensitivity analysis and grayscale-trained comparisons supported the usefulness of chromatic information, while the benefits of histogram integration with grayscale inputs indicated that its contribution was not limited to color information. However, all evaluated models remained sensitive to strong simulated color-temperature shifts, highlighting the importance of white-balance control.

Despite these promising results, several limitations remain. First, the experiments were conducted using images collected from a single experimental laying hen house, with cage-level laying-status labels that do not capture within-cage individual variation. Performance declined substantially on previously unseen cages, and generalization across different farms remains unverified. Second, further validation is required under more diverse commercial environments, including different farm layouts, camera viewpoints, cage structures, occlusion patterns, and background complexity. Third, the current evaluation was limited to a single laying hen breed, and generalization to other breeds with different pigmentation or morphological characteristics should be investigated. Fourth, although the end-to-end evaluation confirmed that the advantage of CHIC is maintained when the head and leg regions are localized automatically, the full-pipeline performance was limited by detection coverage and part assignment, and detection quality under occlusion, pose variation, and complex backgrounds in commercial environments remains to be improved.

Future work will focus on validating CHIC under more diverse commercial poultry-house conditions, including different farm layouts, cage structures, camera viewpoints, occlusion patterns, and breeds. To address the detection bottleneck identified in the end-to-end evaluation, the detector will be improved for robust localization of head and leg regions under occlusion and pose variation, and multi-session acquisition with individual-level partitioning and independently verified individual laying records will be pursued to validate generalization to unseen hens. On-device latency and deployment performance of the full detection–classification pipeline will also be evaluated on resource-constrained edge devices to assess the practical feasibility of real-time poor-laying hen monitoring in precision livestock farming environments.

## Figures and Tables

**Figure 1:**
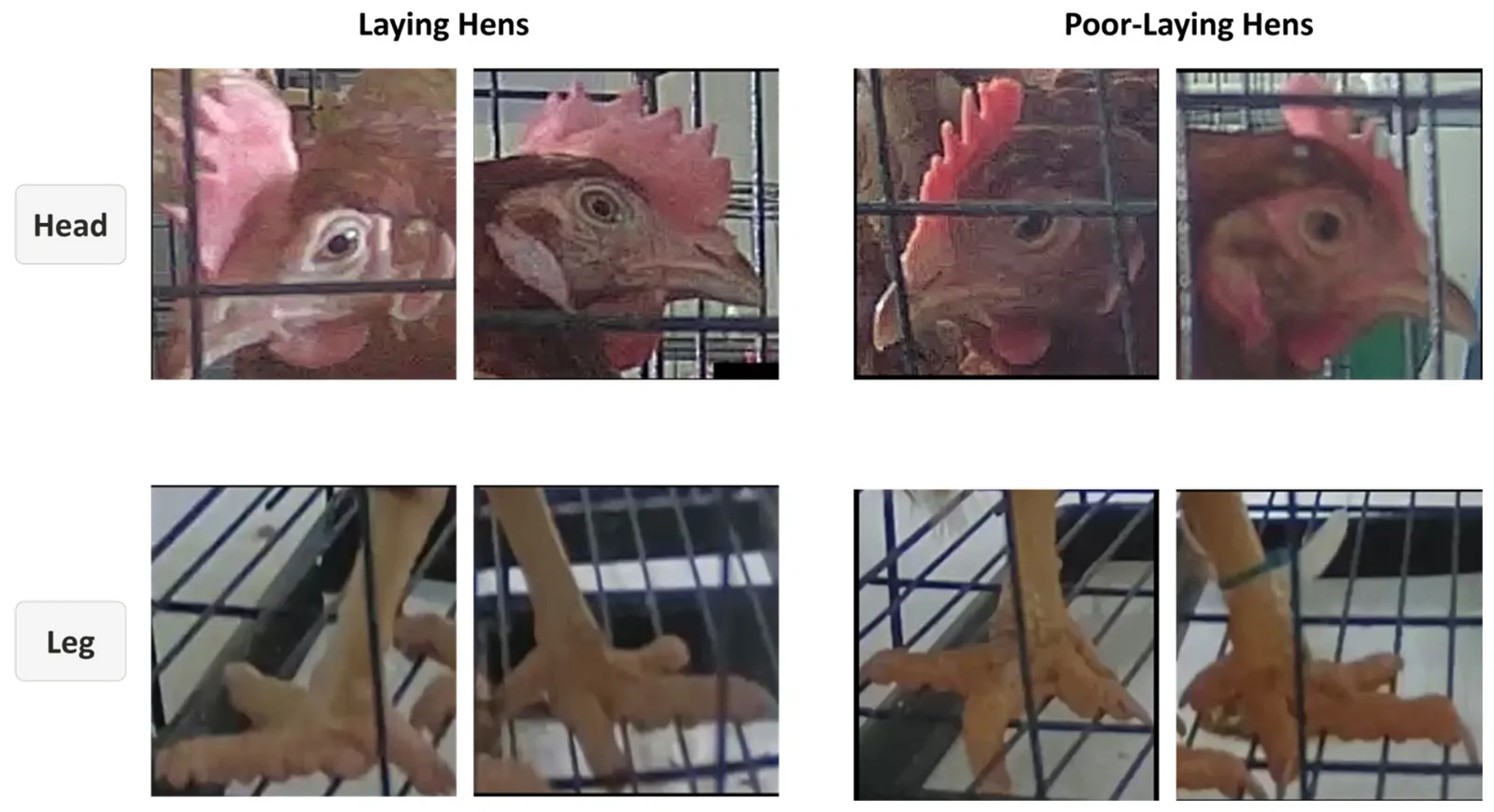
Representative external appearances of laying and poor-laying Hy-Line Brown hens in the head and leg regions.

**Figure 2:**
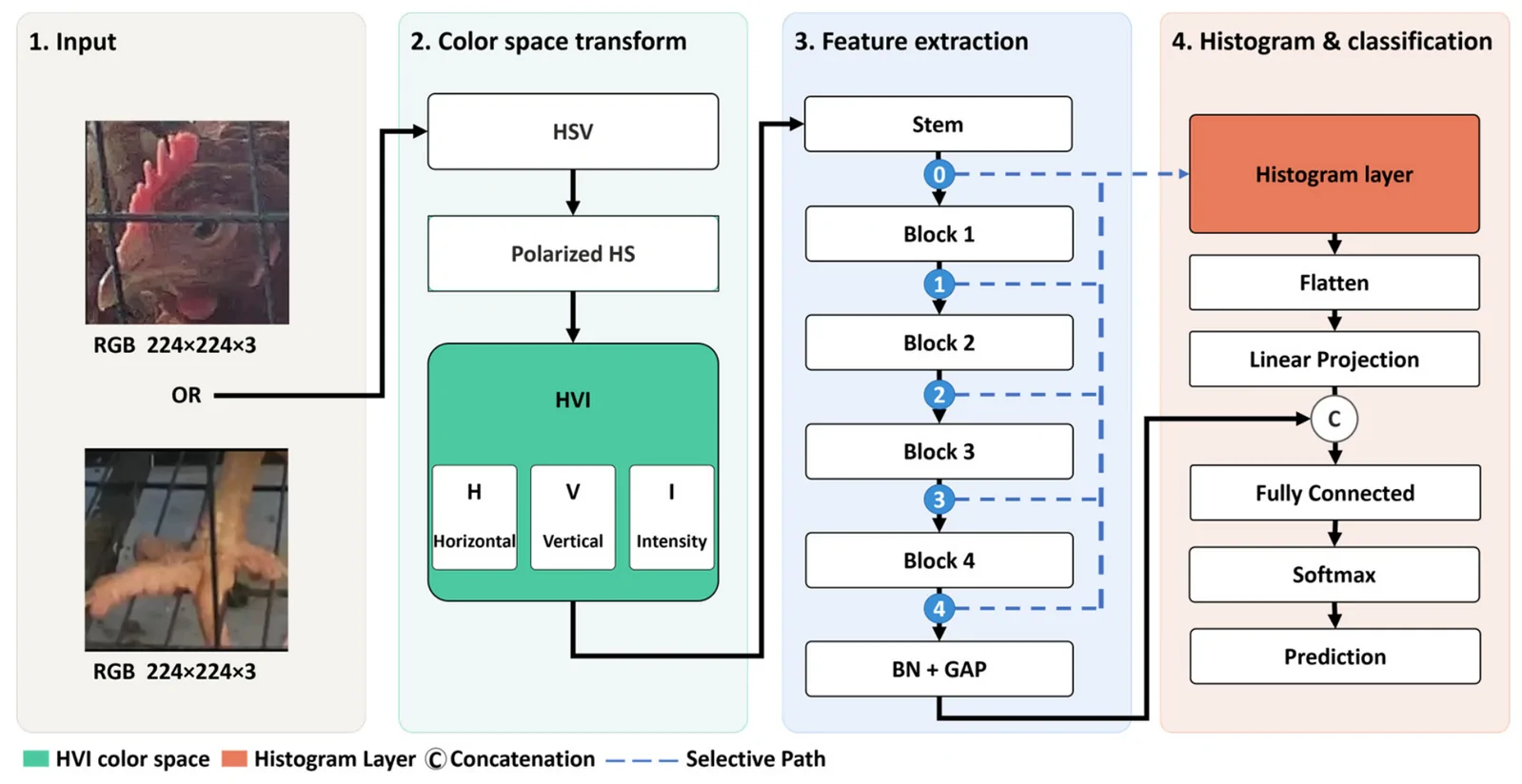
Overall architecture of the proposed CHIC network.

**Figure 3:**
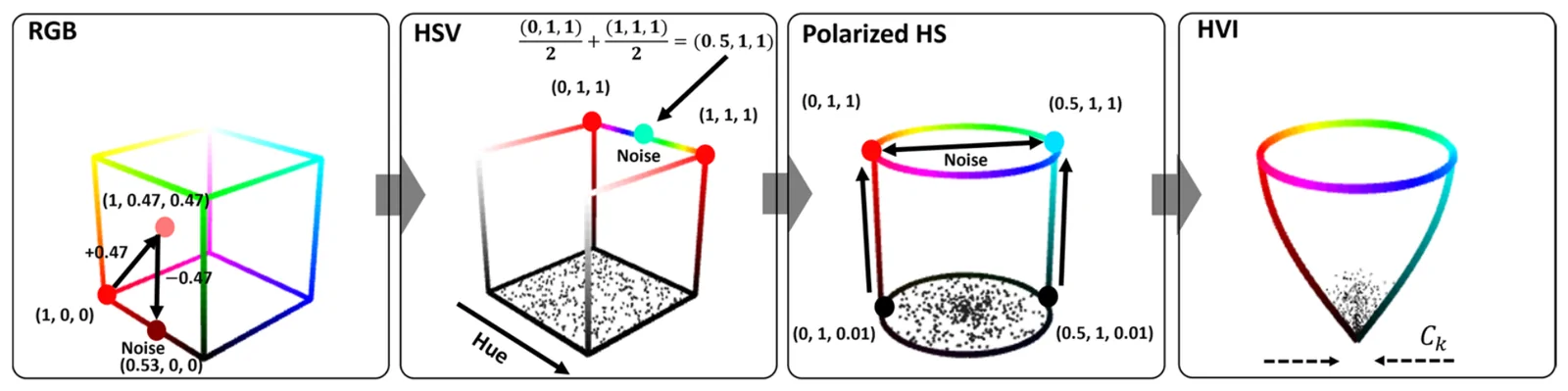
HVI color–space transformation process. Colored outlines depict color variation, red and cyan markers in HSV illustrate hue discontinuity, and black dots represent low-intensity noise.

**Figure 4:**
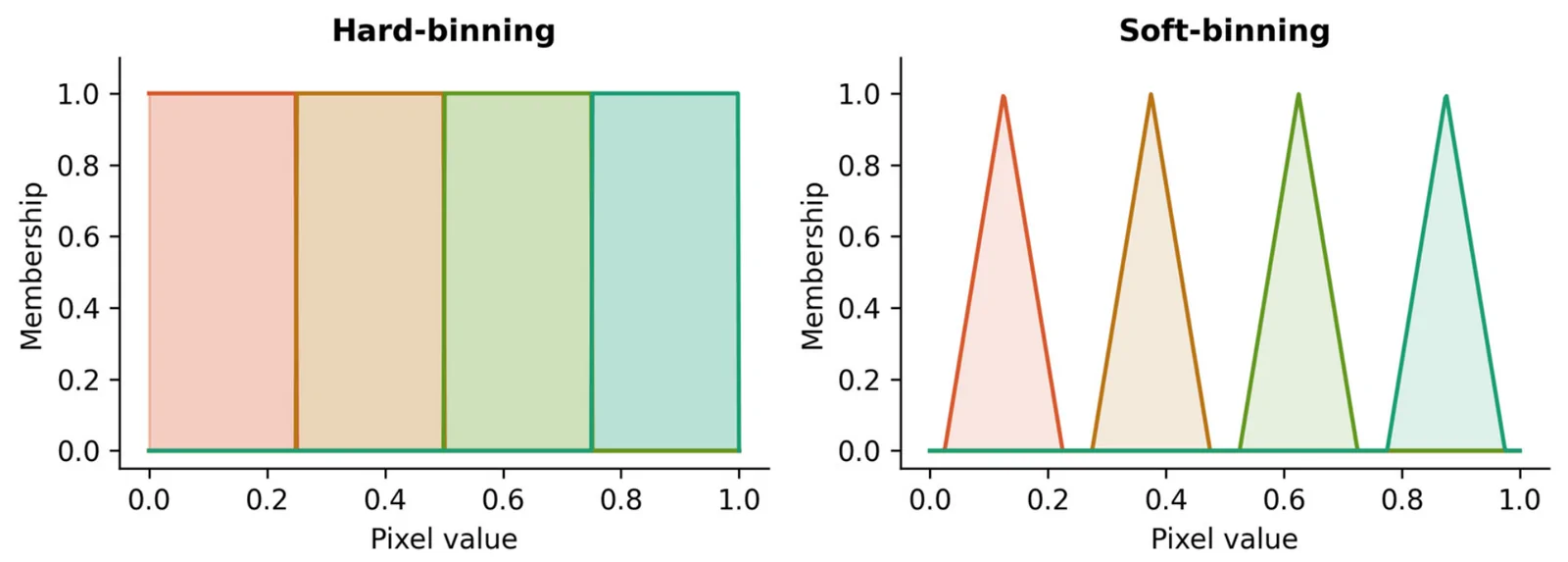
Comparison of hard-binning and soft-binning membership functions.

**Figure 5:**
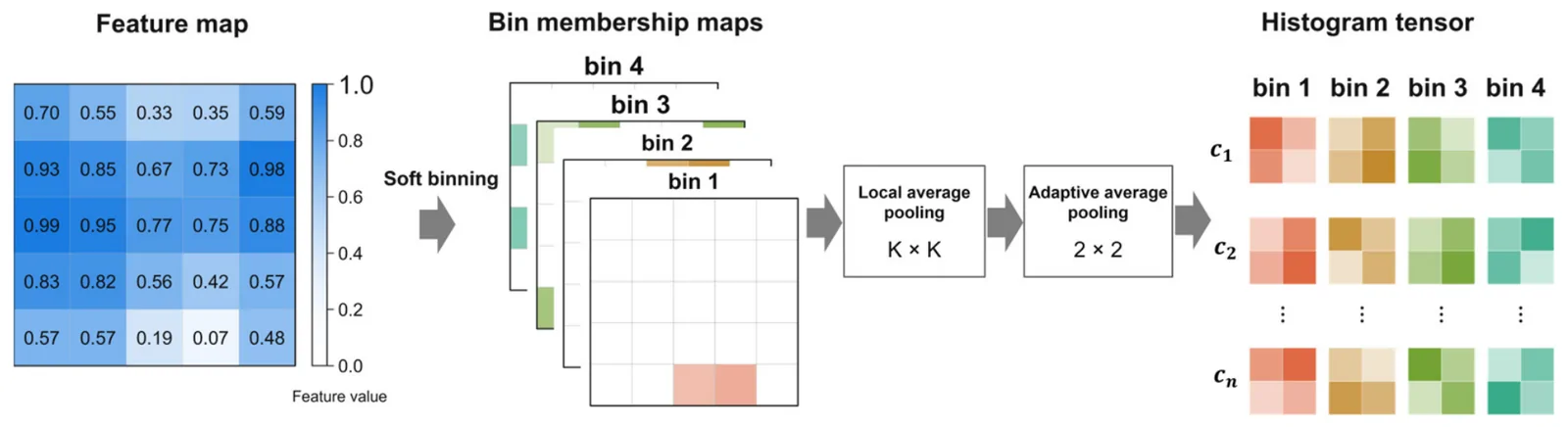
Computation pipeline of the differentiable histogram layer. Colors distinguish histogram bins, while darker shades schematically represent larger pooled soft-membership responses.

**Figure 6:**
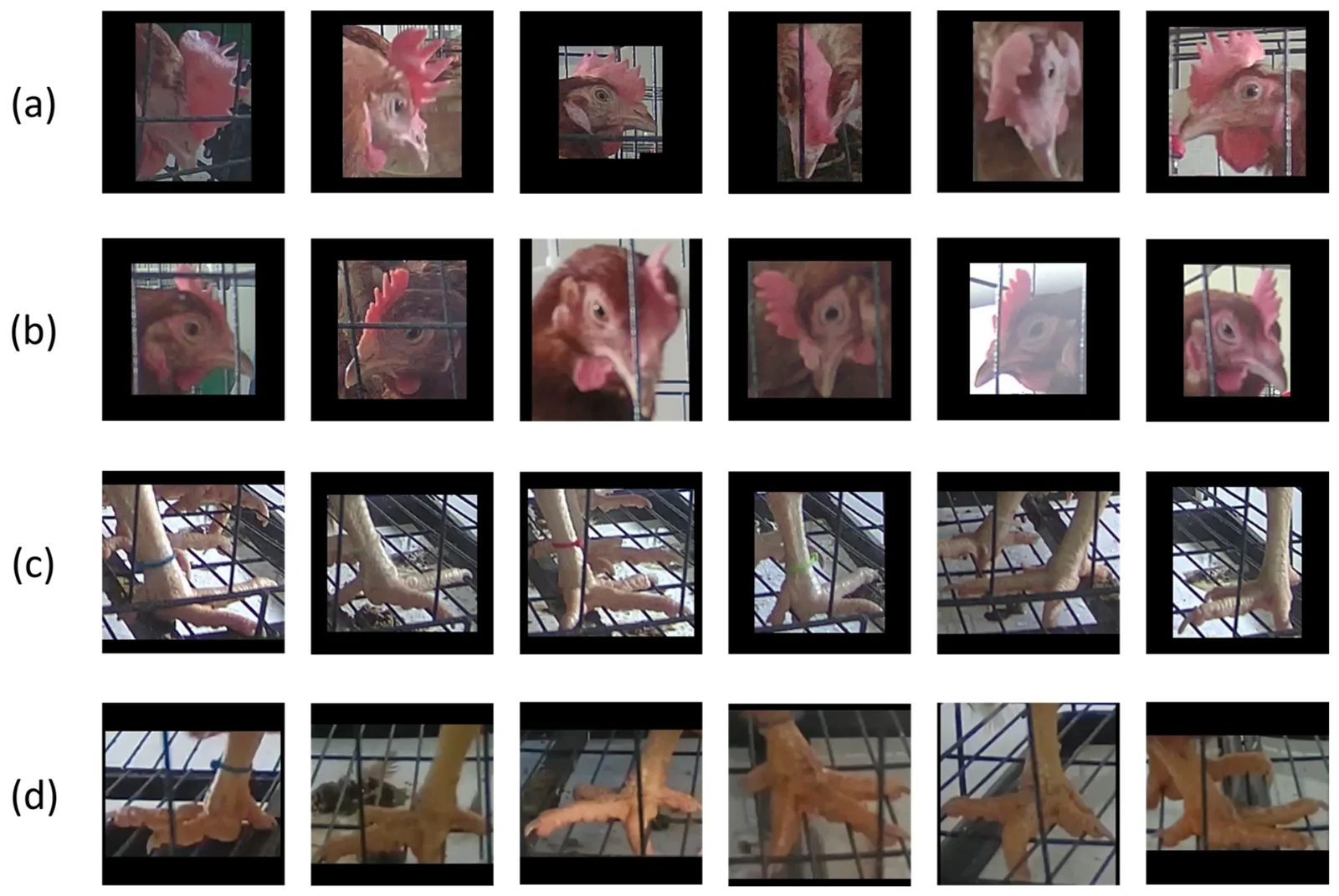
Representative patches of the four classes in the dataset: (**a**) laying head, (**b**) poor-laying head, (**c**) laying leg, and (**d**) poor-laying leg. Patches are shown as they are input to the network, with letterbox padding to 224 × 224 pixels.

**Figure 7:**
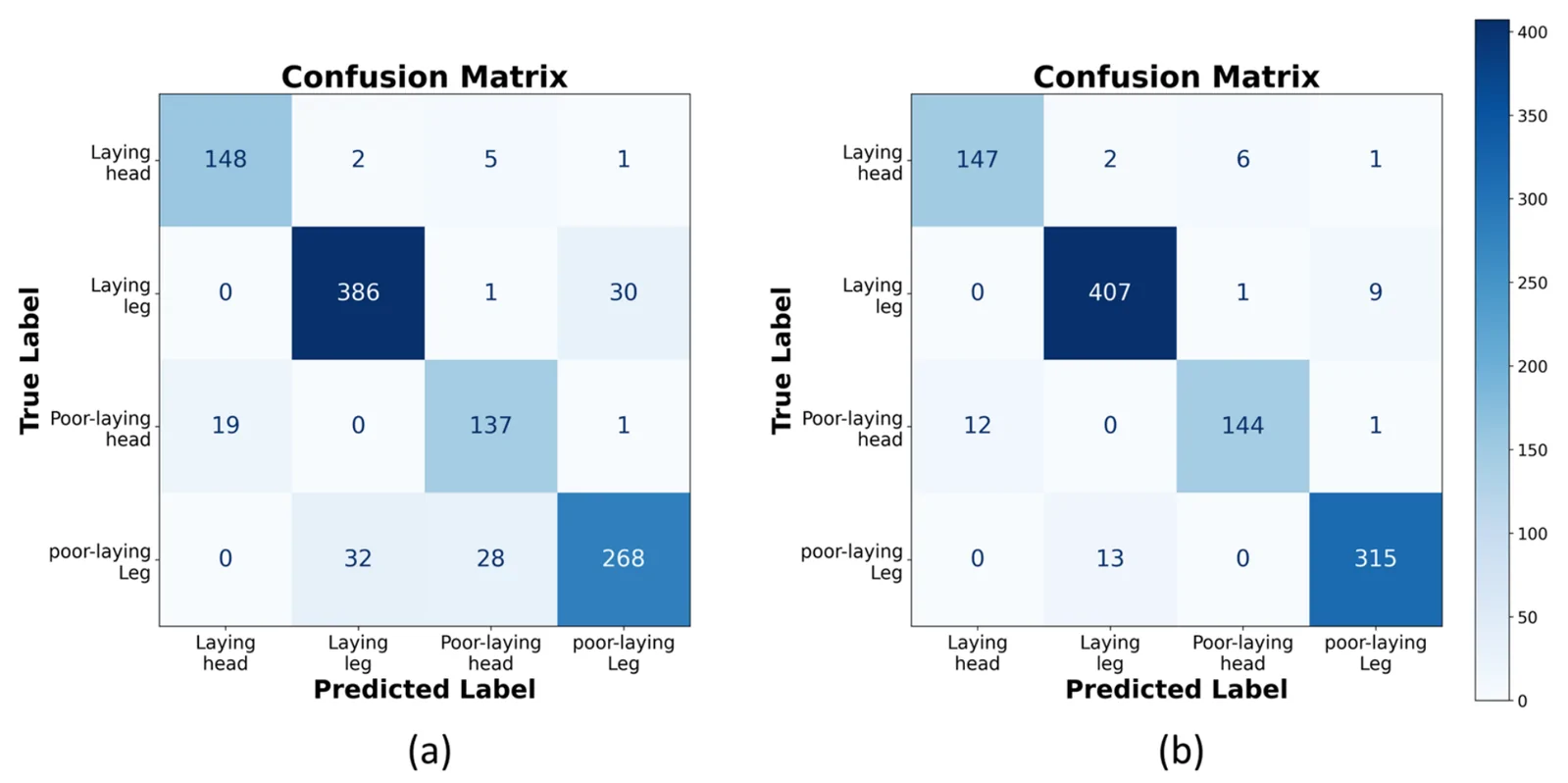
Confusion matrices on the test set for (**a**) MobileNetV4-S and (**b**) CHIC. CHIC reduces both laying-status confusion and head–leg region confusion compared with MobileNetV4-S.

**Figure 8:**
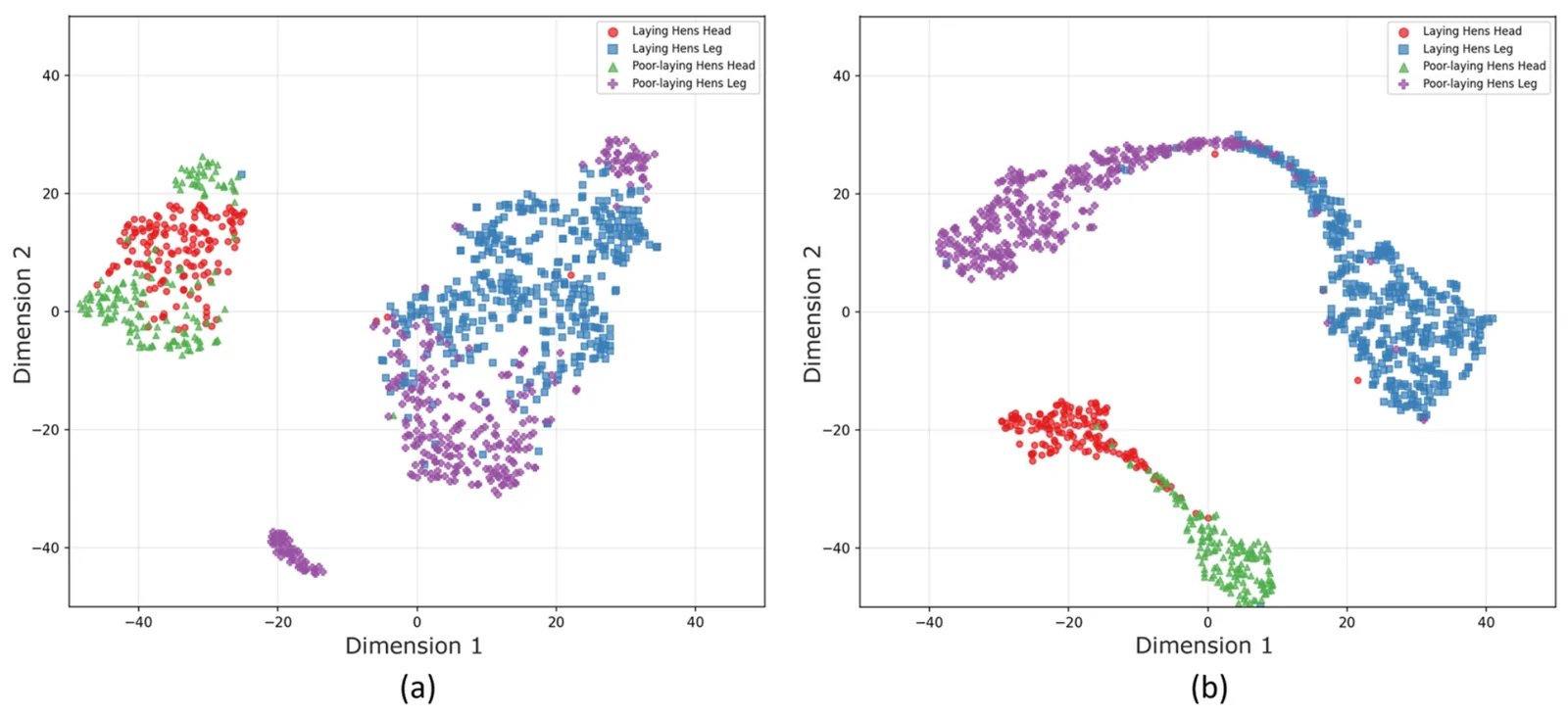
t-SNE visualization of feature representations extracted from (**a**) MobileNetV4-S and (**b**) CHIC. Axes represent the two t-SNE dimensions in arbitrary units.

**Figure 9:**
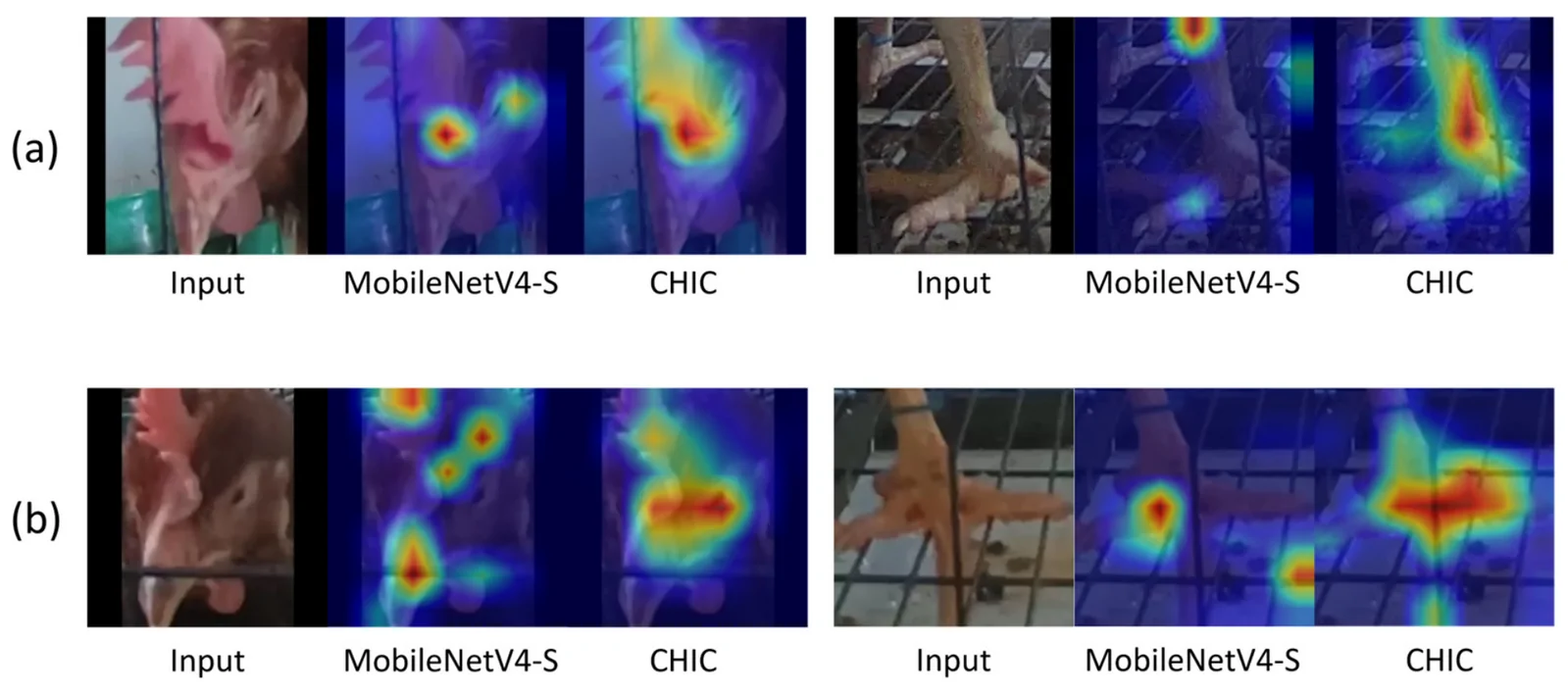
Grad-CAM visualizations of MobileNetV4-S and CHIC: (**a**) head and leg images of laying hens; (**b**) head and leg images of poor-laying hens. CHIC shows more concentrated activation within the head and leg regions than MobileNetV4-S in the examples shown.

**Figure 10:**
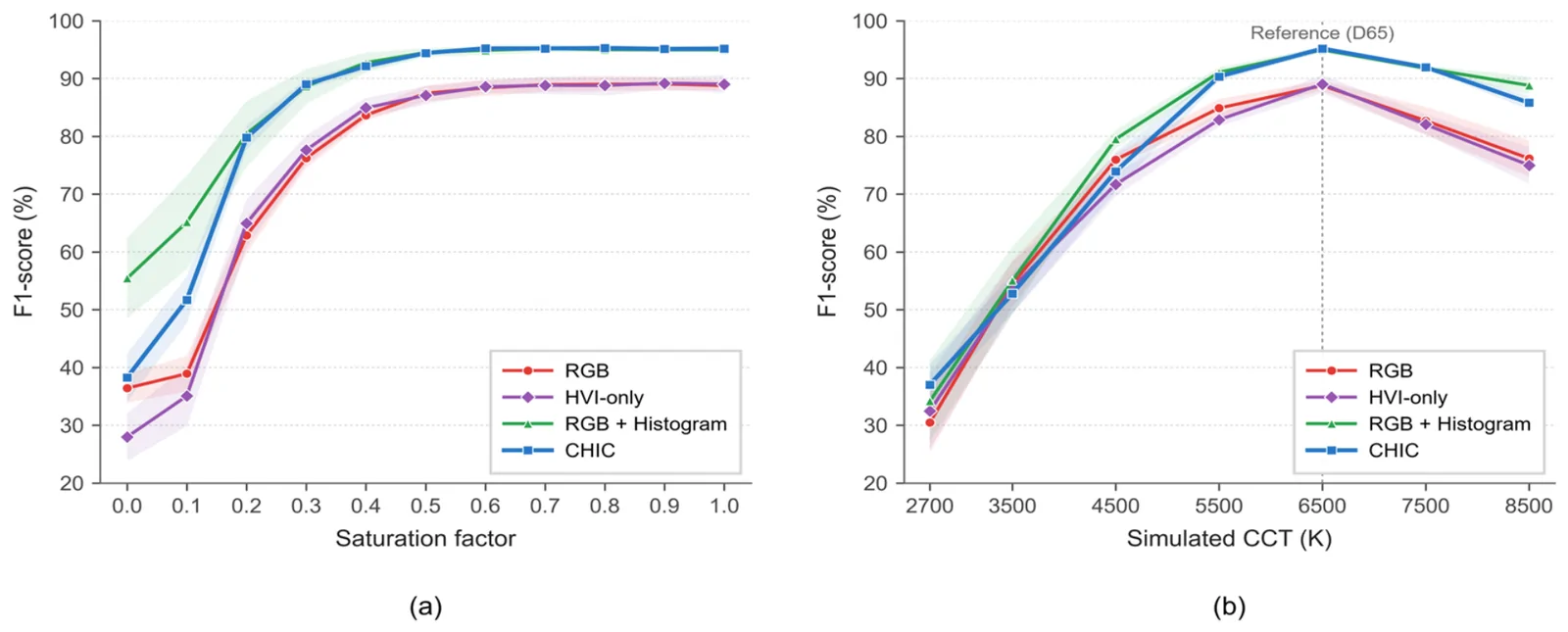
Sensitivity to chromatic perturbations: (**a**) saturation factor and (**b**) simulated CCT for the RGB baseline, HVI-only, RGB + Histogram, and CHIC. Lines represent mean F1-scores, and shaded bands indicate ± one standard deviation over five runs. In (**b**), 6500 K denotes the untransformed test images, treated as the D65 reference white.

**Figure 11:**
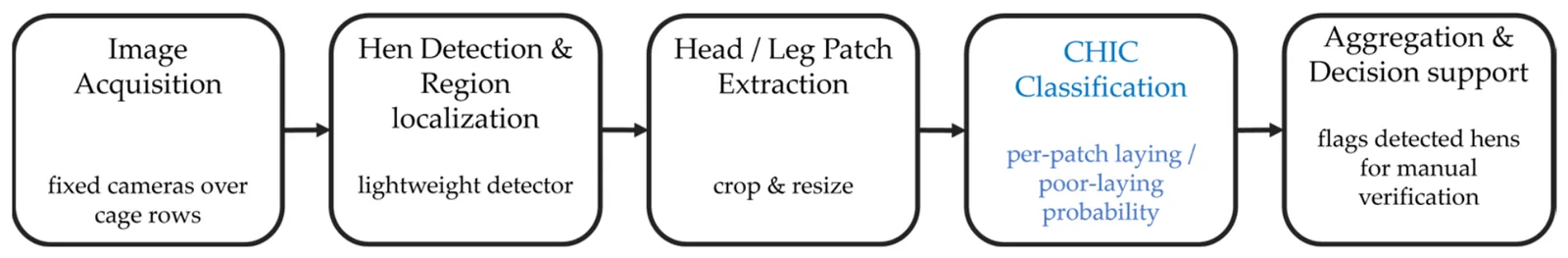
Deployment pipeline of the proposed method in a commercial caged house. Blue denotes the proposed CHIC classifier.

**Table 1:** Dataset composition for the four-class poor-laying hen identification task.

Class	Train	Validation	Test	Total
Laying head	1299	165	156	1620
Laying leg	3239	386	417	4042
Poor-laying head	1180	146	157	1483
Poor-laying leg	2725	325	328	3378
Total	8443	1022	1058	10,523

**Table 2:** Comparison of classification performance, parameter count, and inference latency for MobileNetV4-S baselines, HVI transformation, and histogram integration. Performance metrics are reported as mean ± standard deviation across ten training runs using different development-data folds, with each run evaluated on the same fixed test set.

Model	Head F1-Score (%)	Leg F1-Score (%)	Head ROC-AUC (%)	Leg ROC-AUC (%)	F1-Score (%)	Params (M)	Latency (ms/Image)
MobileNetV4-S (width 1.0)	91.46 ± 2.30	95.16 ± 1.04	98.77 ± 0.38	98.61 ± 0.60	93.03 ± 1.24	2.50	17.61 ± 1.26
MobileNetV4-S (width 0.5)	88.44 ± 2.54	90.53 ± 2.24	95.62 ± 1.11	96.55 ± 1.99	89.21 ± 1.86	0.96	16.93 ± 0.56
+ HVI Transform	90.01 ± 1.22	88.68 ± 2.06	96.08 ± 1.38	95.80 ± 1.52	88.58 ± 0.98	0.96	19.97 ± 0.66
+ Histogram Layer	92.21 ± 1.54	96.06 ± 0.93	97.40 ± 0.67	99.26 ± 0.18	94.07 ± 0.75	2.69	21.87 ± 0.82

**Table 3:** Region-specific binary laying-status classification. Performance metrics are reported as mean ± standard deviation over five runs with different random seeds.

Region	Model	Accuracy (%)	Precision (%)	Recall (%)	F1-Score (%)	ROC- AUC (%)
Head	MobileNetV4-S	89.27 ± 0.92	90.68 ± 1.39	87.64 ± 2.41	89.11 ± 1.04	96.09 ± 0.84
	CHIC	94.50 ± 0.61	95.46 ± 0.96	93.50 ± 1.38	94.46 ± 0.64	98.27 ± 0.37
Leg	MobileNetV4-S	89.83 ± 1.52	88.47 ± 2.27	88.48 ± 2.83	88.44 ± 1.76	95.69 ± 1.74
	CHIC	96.05 ± 0.35	95.34 ± 1.02	95.73 ± 0.37	95.53 ± 0.37	99.01 ± 0.14

**Table 4:** Quantitative attention alignment between Grad-CAM maps and the segmented head/leg regions on the test set.

Class	Model	Norm. RoI Energy (%)	Pointing Game (%)	Pixel ROC- AUC (%)	Norm. IoU@20 (%)
Overall	MobileNetV4-S	5.70	41.91	53.83	9.43
	CHIC	28.15	69.38	68.71	35.78
Laying head	MobileNetV4-S	25.26	75.91	63.77	35.33
	CHIC	48.23	91.67	82.19	67.72
Laying leg	MobileNetV4-S	0.41	30.46	51.31	2.73
	CHIC	18.33	65.47	65.24	21.00
Poor- laying head	MobileNetV4-S	8.23	54.14	55.33	14.49
	CHIC	45.13	78.34	73.03	61.39
Poor- laying leg	MobileNetV4-S	1.91	34.45	51.60	3.22
	CHIC	22.96	59.45	64.65	27.13

**Table 5:** Performance comparison of different backbone networks with baseline and CHIC architectures. Performance metrics are reported as mean ± standard deviation over five runs with different random seeds.

Backbone	Architecture	Bin	Kernel	Stage	F1-Score (%)	Params (M)	FLOPs (G)	Latency (ms/Image)
MobileNetV4-S	Baseline	-	-	-	88.78 ± 0.85	0.96	0.13	16.93 ± 0.56
	CHIC	2	3	4	95.17 ± 0.49	2.69	0.20	21.87 ± 0.82
EfficientNetV2-b0	Baseline	-	-	-	97.42 ± 0.42	5.86	1.47	34.76 ± 2.20
	CHIC	2	3	4	96.45 ± 0.52	7.54	1.48	36.34 ± 1.16
GhostNetV3-100	Baseline	-	-	-	96.56 ± 0.46	6.85	0.87	114.15 ± 1.34
	CHIC	2	3	4	96.28 ± 0.25	8.52	1.00	119.44 ± 1.13

**Table 6:** Component-level ablation of the HVI transformation and differentiable histogram layer, and comparison with alternative color spaces. Performance metrics are reported as mean ± standard deviation over five runs with different random seeds.

Backbone	Color Space	Histogram Layer	Bin	Kernel	Stage	F1-Score (%)	Params (M)
MobileNetV4-S	RGB	No	-	-	-	88.78 ± 0.85	0.96
	HSV	No	-	-	-	88.03 ± 1.30	0.96
	CIELAB	No	-	-	-	88.90 ± 1.63	0.96
	YCbCr	No	-	-	-	88.97 ± 1.23	0.96
	HVI	No	-	-	-	89.04 ± 1.53	0.96
	Grayscale	No	-	-	-	81.88 ± 0.68	0.96
	RGB	Yes	2	3	4	94.97 ± 0.50	2.69
	HSV	Yes	2	3	4	93.89 ± 0.73	2.69
	CIELAB	Yes	2	3	4	95.37 ± 0.59	2.69
	YCbCr	Yes	2	3	4	95.21 ± 0.59	2.69
	Grayscale	Yes	2	3	4	90.16 ± 0.92	2.69
	HVI	Yes	2	3	4	95.17 ± 0.49	2.69

**Table 7:** Robustness under illumination-related perturbations. Performance metrics are reported as mean ± standard deviation over five runs with different random seeds.

Model	Original (%)	Brightness (%)	Contrast (%)	Gamma (%)	Average Drop (pp)
MobileNetV4-S	88.78 ± 0.85	83.54 ± 1.49	86.03 ± 1.06	76.51 ± 2.57	6.75
HVI- only	89.04 ± 1.53	85.59 ± 1.62	86.59 ± 1.78	78.63 ± 2.83	5.43
RGB- histogram	94.97 ± 0.50	92.49 ± 0.59	93.28 ± 0.54	83.99 ± 1.06	5.06
CHIC	95.18 ± 0.48	91.94 ± 0.42	93.25 ± 0.22	85.23 ± 0.57	5.04

**Table 8:** Sensitivity analysis of histogram layer configurations across different backbone stages and kernel sizes. Performance metrics are reported as mean ± standard deviation over five runs with different random seeds.

Model	Bin	Kernel	Stage	F1-Score (%)	Average Pooling (%)	Histogram Pooling (%)	Params (M)
CHIC	2	11	0	94.48 ± 0.35	77.01 ± 0.85	91.08 ± 0.88	2.61
	2	9	1	94.99 ± 0.57	77.69 ± 1.97	90.79 ± 0.76	2.61
	2	7	2	94.30 ± 0.62	82.25 ± 1.47	91.06 ± 0.80	2.61
	2	5	3	94.96 ± 0.68	93.65 ± 0.77	93.91 ± 0.91	2.61
	2	3	4	95.17 ± 0.49	95.30 ± 0.58	95.36 ± 0.40	2.69

**Table 9:** Sensitivity analysis of the histogram layer with different numbers of bins. Performance metrics are reported as mean ± standard deviation over five runs with different random seeds.

Model	Bin	Kernel	Stage	Valid F1-Score (%)	Test F1-Score (%)	Params (M)
CHIC	2	3	4	95.51 ± 0.64	95.17 ± 0.49	2.69
	4	3	4	95.38 ± 0.31	95.32 ± 0.56	2.64
	8	3	4	95.13 ± 0.48	95.10 ± 0.45	2.62
	16	3	4	95.61 ± 0.27	95.00 ± 0.20	2.61

**Table 10:** Comparison of mean–standard-deviation pooling with the histogram layer in classification performance. Performance metrics are reported as mean ± standard deviation over five runs with different random seeds.

Model	Head F1-Score (%)	Leg F1-Score (%)	Head ROC-AUC (%)	Leg ROC-AUC (%)	F1-Score (%)
RGB-Mean-Std	95.38 ± 1.43	96.33 ± 0.31	98.69 ± 0.43	99.04 ± 0.20	95.96 ± 0.77
HSV-Mean-Std	93.81 ± 0.85	94.78 ± 0.73	98.24 ± 0.31	98.50 ± 0.50	94.41 ± 0.72
YCbCr-Mean-Std	94.95 ± 0.86	96.27 ± 0.36	98.7 ± 0.52	98.94 ± 0.30	95.79 ± 0.38
CIELAB-Mean-Std	95.38 ± 0.94	96.10 ± 0.72	98.67 ± 0.37	99.22 ± 0.17	95.76 ± 0.69
HVI-Mean-Std	94.34 ± 0.53	95.91 ± 0.62	98.45 ± 0.56	99.22 ± 0.17	95.20 ± 0.40
CHIC	94.46 ± 0.64	95.53 ± 0.37	98.27 ± 0.37	99.01 ± 0.14	95.17 ± 0.49

**Table 11:** Classification performance under three-fold cage-disjoint evaluation. Performance metrics are reported as mean ± standard deviation across three folds.

Model	Valid F1-Score (%)	Test F1-Score (%)	Head F1-Score (%)	Leg F1-Score (%)	Head ROC-AUC (%)	Leg ROC-AUC (%)
MobileNetV4-S	89.98 ± 2.28	57.05 ± 9.13	56.57 ± 26.73	45.35 ± 23.73	74.10 ± 7.44	52.83 ± 9.49
HVI- only	89.55 ± 2.66	59.08 ± 9.12	59.71 ± 20.99	45.46 ± 28.08	75.12 ± 4.43	54.37 ± 15.76
RGB- histogram	94.61 ± 1.44	56.57 ± 7.63	60.29 ± 15.13	44.34 ± 33.06	71.93 ± 5.50	46.96 ± 18.91
CHIC	94.82 ± 0.98	60.51 ± 8.71	63.43 ± 14.15	49.69 ± 30.49	75.60 ± 4.45	55.16 ± 18.98

**Table 12:** Cage-identity probe accuracy and classification F1-scores before and after background masking or letterbox-padding removal. Results are reported as mean ± standard deviation over five runs with different random seeds.

Model	F1-Score (%)	Cage-ID Probe (%)	Background Removed F1-Score (%)	Padding Removed F1-Score (%)
MobileNetV4-S (width 0.5)	88.78 ± 0.85	76.7 ± 1.3	69.33 ± 4.01	84.66 ± 1.59
HVI Transform	89.04 ± 1.53	76.0 ± 1.7	73.05 ± 3.27	84.99 ± 1.59
RGB Histogram	94.97 ± 0.50	85.3 ± 0.4	75.93 ± 4.40	91.96 ± 0.53
CHIC	95.17 ± 0.49	82.7 ± 0.8	79.19 ± 1.93	92.38 ± 0.45

**Table 13:** Classification performance of color moments and color histograms under different padding and background conditions. Performance metrics are reported as mean ± standard deviation over five runs with different random seeds.

Feature	Color Space	Dimension	w/Padding F1-Score (%)	w/o Padding F1-Score (%)	w/o Background F1-Score (%)
Color moment	RGB	9	59.15 ± 0.40	69.46 ± 0.77	73.46 ± 0.29
Color moment	HSV	9	65.08 ± 0.44	75.02 ± 0.23	75.43 ± 0.45
Color moment	CIELAB	9	72.11 ± 0.36	75.79 ± 0.44	73.81 ± 0.55
Color moment	YCbCr	9	73.12 ± 0.87	75.61 ± 0.44	73.62 ± 0.29
Color moment	HVI	9	76.88 ± 0.21	81.20 ± 0.62	75.26 ± 0.38
Color histogram	RGB	48	73.46 ± 0.56	77.23 ± 0.22	77.18 ± 0.35
Color histogram	HSV	48	84.83 ± 0.70	84.48 ± 0.36	79.47 ± 0.59
Color histogram	CIELAB	48	79.03 ± 0.7	80.75 ± 0.38	77.87 ± 0.32
Color histogram	YCbCr	48	78.86 ± 0.53	79.84 ± 0.47	77.80 ± 0.65
Color histogram	HVI	48	81.05 ± 0.64	83.06 ± 0.14	80.08 ± 0.39

**Table 14:** Comparison of classification performance and parameter count between CHIC and existing classification models. Performance metrics are reported as mean ± standard deviation over five runs with different random seeds.

Model	Head F1-Score (%)	Leg F1-Score (%)	F1-Score (%)	Params (M)	FLOPs (G)	Latency (ms/Image)
CHIC	94.46 ± 0.64	95.53 ± 0.37	95.17 ± 0.49	2.69	0.20	21.87 ± 0.82
MobileNetV3-S [14]	89.27 ± 2.91	91.08 ± 2.77	90.50 ± 2.54	1.52	0.11	17.32 ± 0.51
ConvNext-Tiny [22]	93.41 ± 0.52	94.55 ± 0.59	94.20 ± 0.52	27.82	8.99	17.98 ± 1.82
ConvNext-Tiny + SE [22]	93.03 ± 0.59	92.28 ± 2.39	92.99 ± 1.10	28.24	8.99	27.58 ± 0.95

**Table 15:** End-to-end performance of the detection and classification pipeline.

Pipeline	E2E Success Rate (%)	mAP@0.5 (%)	mAP@0.5:0.95 (%)	Hen-Level E2E Success Rate (%)	Hen-Level Classification Accuracy (%)
YOLO26n + MobileNetV4-S	77.79	78.15	47.51	75.76	94.34
YOLO26n + CHIC	82.61	82.88	50.27	79.00	98.38

## Data Availability

The data supporting the findings of this study are not publicly available due to confidentiality and data-sharing restrictions associated with the funded research project. Requests for access should be directed to the corresponding author and will be considered subject to prior authorization from the participating institutions.

## Abbreviations

The following abbreviations are used in this manuscript:

CHIC	Color-Histogram Integrated Classifier
CNN	Convolutional neural network
GAP	Global average pooling
Grad-CAM	Gradient-weighted class activation mapping
HSV	Hue, saturation and value
HVI	Horizontal, vertical and intensity
PLF	Precision livestock farming
RGB	Red, Green and blue
t-SNE	t-distributed stochastic neighbor embedding
